# Magnetic Resonance Imaging Features of Hepatic Hydatid Disease: A Pictorial Review with Emphasis on Atypical Presentations and Differential Diagnosis

**DOI:** 10.3390/diagnostics16091304

**Published:** 2026-04-27

**Authors:** Jelena Djokic Kovač, Aleksandra Đikić-Rom, Aleksandra Janković, Nikica Grubor, Aleksandra Đurić-Stefanović, Aleksandar Bogdanović, Milica Mitrović, Ognjan Skrobić, Andrija Antić, Đorđe Knežević, Goran Đuričić, Predrag Zdujić, Nemanja Bidžić

**Affiliations:** 1 Department for Digestive Radiology, Center for Radiology, University Clinical Center of Serbia, 11000 Belgrade, Serbia; jankovicmalex@gmail.com (A.J.); aleksandra.djuricstefanovic@gmail.com (A.Đ.-S.); dr_milica@yahoo.com (M.M.); 2 School of Medicine, University of Belgrade, 11000 Belgrade, Serbia; gruborn13@gmail.com (N.G.); aleksandarbogdanovic81@yahoo.com (A.B.); skrobico@gmail.com (O.S.); andrija.antic@kcs.ac.rs (A.A.); djordje.knezevic@kcs.ac.rs (Đ.K.); gorandjuricic@gmail.com (G.Đ.); pedjazdujic@yahoo.com (P.Z.); nemanja.bidzic@kcs.ac.rs (N.B.); 3 Department of Pathology, Clinic for Digestive Surgery, University Clinical Center of Serbia, 11000 Belgrade, Serbia; aleksandra.djikic.rom@gmail.com; 4 Clinic for Digestive Surgery—First Surgical Clinic, University Clinical Center of Serbia, 11000 Belgrade, Serbia; 5 Department of Radiology, University Children’s Hospital, School of Medicine, University of Belgrade, 11000 Belgrade, Serbia

**Keywords:** hepatic echinococcosis, cystic echinococcosis, alveolar echinococcosis, magnetic resonance imaging

## Abstract

Hepatic echinococcosis, caused by *Echinococcus* species, remains a significant global health concern, with cystic echinococcosis (CE) being widespread and alveolar echinococcosis (AE) representing a rarer but more aggressive form. CE generally demonstrates characteristic imaging features, allowing straightforward diagnosis, whereas atypical presentations can closely mimic other hepatic lesions, leading to diagnostic uncertainty. AE poses an even greater diagnostic challenge due to its infiltrative, tumor-like growth pattern. Magnetic resonance imaging (MRI), with its superior soft-tissue contrast and multiplanar capabilities, plays a crucial role in the evaluation of AE and atypical CE cases. This pictorial review summarizes MRI features of hepatic echinococcosis, detailing both typical and atypical appearances, and emphasizes key criteria for differentiating hydatid cysts from other cystic or solid hepatic lesions. By consolidating imaging findings and discussing relevant differential diagnoses, this review aims to improve diagnostic accuracy, guide clinical management, and increase radiologists’ awareness of echinococcosis in both endemic and non-endemic regions.

## 1. Introduction

Echinococcosis is a zoonosis caused by the larval stage of tapeworms from the genus Echinococcus, predominantly *Echinococcus granulosus* and *Echinococcus multilocularis* [1]. *E. granulosus* causes cystic echinococcosis (CE), which is widespread and common, whereas *E. multilocularis* causes alveolar echinococcosis (AE), a less frequent but more aggressive form [2]. While CE is generally amenable to treatment, AE carries a poorer prognosis if left untreated, with mortality rates exceeding 90% within 10–15 years. While traditionally endemic to regions like the Mediterranean and South America, global migration has facilitated its spread to previously non-endemic areas [3]. The World Health Organization has included echinococcosis in the list of neglected endemic diseases, aiming for improved control and eventual elimination by 2050 [4].

In most cases, CE demonstrates characteristic radiological features, and diagnosis is straightforward using ultrasound [5]. However, atypical presentations might mimic other liver lesions, and misdiagnosis is not uncommon [6,7]. AE poses a greater diagnostic challenge due to its tumor-like growth and rarity [8]. As liver echinococcosis increasingly appears outside endemic regions, radiologists worldwide must be familiar with both typical and atypical imaging features [9]. Magnetic resonance imaging (MRI), with its superior soft-tissue contrast, is particularly valuable for characterizing these lesions [10]. This manuscript presents a pictorial review of hepatic echinococcosis, emphasizing MRI findings and atypical presentations, with practical guidance for differential diagnosis.

## 2. Pathogenesis and Clinical Presentation

### 2.1. Cystic Echinococcosis

*E. granulosus* is a widely distributed zoonosis and is endemic in Eastern and Southern Europe, Russia, the Middle East, South America, and China in livestock-raising areas [11]. The adult form of *E. granulosus* resides in the intestines of the definitive host (dogs and other canids), where eggs are released from gravid proglottids and excreted in feces [12]. Humans are accidentally infected after ingesting food or water contaminated by the feces of the definitive host [12,13]. Following ingestion of parasite eggs, oncospheres are released into the intestine, and after penetration of the gut wall, they enter portal circulation and reach the liver, which is the most commonly affected organ [14]. Within the liver, oncospheres develop into hydatid cysts, which represent the larval stage of the disease [14]. The wall of CE is composed of three layers, with the inner layer being the germinal membrane or endocyst from which protoscoleces, brood membranes, and daughter cysts are formed [15]. The middle layer or ectocyst is an acellular laminated membrane that allows for the passage of nutrients [15]. The outer layer is the pericyst, which is composed of host fibrotic and granulation tissue [15]. Budding of the germinal membrane gives rise to small vesicles known as brood capsules, which contain numerous protoscoleces, the infective larval forms that may give rise to new adult tapeworms when ingested by the definitive host [14,15]. With further proliferation, the germinal layer may also form daughter cysts, larger vesicular structures possessing their own laminated and germinal layers, capable of producing additional brood capsules and protoscoleces [15]. Daughter cysts contribute to the progressive enlargement and survival of the hydatid cyst. They enable endogenous proliferation of the parasite within the mother cyst and, in the case of rupture, may lead to secondary echinococcosis through dissemination of viable cyst elements [14,16]. When the brood capsules rupture, the protoscoleces are released into the cystic fluid, where they remain viable and form the so-called hydatid sand [15].

Clinical symptoms of CE usually appear late as the cysts grow slowly [13]. It has been shown that most cysts grow up to 1 cm in the first six months and not more than 2–3 cm annually [17]. Thus, the disease can remain asymptomatic for a long period of time [17]. CE is commonly discovered incidentally in many patients [18]. Symptoms occur due to the mass effect on adjacent structures [18]. Patients might complain of weight loss, abdominal discomfort, nausea, and malaise [19]. Compression of the biliary tree, hepatic veins, and portal vein may lead to obstructive jaundice, Budd–Chiari syndrome, and portal hypertension [19,20]. Allergic reactions as an important clinical manifestation of CE may result from spontaneous leakage of antigenic cyst fluid into the circulation, presenting with symptoms such as urticaria, pruritus, eosinophilia, or even anaphylaxis [16,17]. Rupture of the cyst into the biliary ducts, leading to biliary obstruction or cholangitis, might be the initial presentation of the disease. Similarly, rupture into the peritoneal cavity might cause abdominal pain, anaphylaxis, and secondary peritoneal hydatidosis [16,20].

### 2.2. Alveolar Echinococcosis

The endemic regions for AE are in the Northern Hemisphere, including North America, China, and Central Europe [21]. However, the increase in the population of infected foxes and their broader geographic distribution has led to an increased incidence in European countries such as France, Switzerland, Germany, Austria, and the Baltic region [21,22]. In the case of *Echinococcus multilocularis*, the definitive hosts are foxes and dogs, while the intermediate hosts are rodents [23]. As in CE, humans are infected accidentally by consuming contaminated fruits or vegetables [23]. In the intestines of the intermediate host, the oncospheres are released from eggs [23]. After penetrating the intestinal wall, they enter the portal circulation and consequently reach the liver [23,24]. In contrast to cystic echinococcosis, alveolar echinococcosis exhibits infiltrative growth, similarly to malignant liver tumors [25]. AE is characterized by the formation of complex, alveolar-like structures consisting of multiple vesicles ranging from 5 mm to 3 cm in size [23]. These vesicles contain viable protoscolices, which can disseminate to neighboring tissues or even metastasize to distant organs [25,26]. The wall of the vesicles consists of two layers: the inner layer is the germinal membrane, and the outer layer is the laminated acellular membrane, which is resistant to immune responses, allowing the parasite to escape host defense mechanisms [24]. Unlike CE, AE does not form a pericyst, which may explain the increased aggressiveness of AE [26]. Over time, the vesicles grow and cause progressive liver damage, including fibrosis, necrosis, and, in advanced stages, cirrhosis [26]. The host’s immune response leads to the formation of fibrosis and granulation tissue around the lesion, which represents the solid component of AE [25,26]. Due to poor vascularization, liquefactive necrosis is commonly observed in the center of large lesions [25]. An exuberant fibrosis leads to obstruction of nearby major vessels and bile ducts with consequent atrophy of liver parenchyma surrounding the lesion, accompanied by capsular retraction [25]. In addition, AE lesions have a propensity to infiltrate hilar structures, leading to severe morphological and functional impairment [26].

Similar to CE, AE lesions also grow very slowly, with clinical manifestations usually occurring several years after infestation [23,24]. Weight loss and fatigue are early symptoms, while obstructive jaundice and portal hypertension develop later in the course of the disease, due to the mass effect on biliovascular structures [27]. It has been reported that AE lesions grow faster in immunocompromised patients, with unusual clinical presentations [28]. The equivocal imaging findings in these patients contribute to delayed diagnosis [28]. Radiological imaging is essential for diagnosis and staging, with MRI being the preferred modality due to its superior soft-tissue resolution [29]. Serological tests, particularly ELISA and Western blotting, are crucial for confirming the diagnosis, although they may be negative in some cases [23]. In challenging cases, percutaneous liver biopsy may be necessary for definitive diagnosis [26].

## 3. Imaging Findings of Cystic Echinococcosis

### 3.1. WHO-IWGE Classification of Cystic Echinococcosis

The typical growth pattern in CE is concentric expansion, resulting in a round, cystic lesion that compresses the surrounding liver parenchyma without infiltration [19]. This growth pattern may be explained by the protective role of the pericyst in CE [30]. Specifically, the pericyst, which surrounds the parasitic development within the germinal layer, acts as a barrier that prevents direct invasion of the liver parenchyma [30]. Taking into account that serological tests have low sensitivity in the diagnosis of CE, imaging findings in association with clinical and epidemiological data have a major role in the diagnosis of CE [31]. The World Health Organization Informal Working Group on Echinococcosis (WHO-IWGE) international classification [32] describes six types of CE based on ultrasound imaging findings, which can be correlated with appearances in computed tomography (CT) and MRI [32]. Active forms of echinococcosis include CL (cystic lesion), CE1, and CE2 types. CE3 is transitional, while CE4 and CE5 are inactive stages [32].

#### 3.1.1. CL Stage

**Figure 1:**
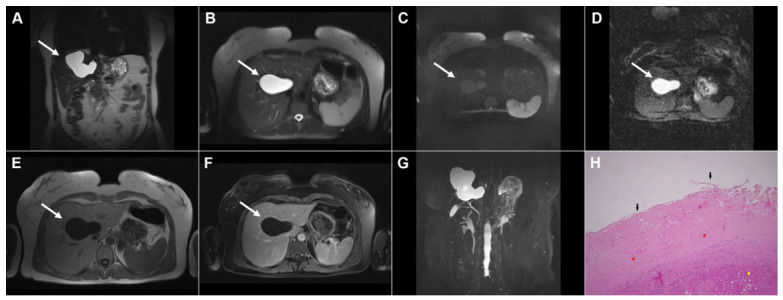
Images of a 33-year-old woman with cystic echinococcosis, WHO stage CL. Coronal T2-weighted image (**A**) demonstrates an irregularly shaped, well-circumscribed cystic lesion in liver segment VIII (*arrow*). Axial T2-weighted fat-suppressed image (**B**) shows an elongated cystic lesion with an imperceptible wall and homogeneously hyperintense fluid content (*arrow*). Diffusion-weighted image (**C**) and the corresponding ADC map (**D**) reveal no restricted diffusion within the lesion (*arrow*). The cyst (*arrow*) appears hypointense in the T1-weighted image (**E**) and shows no enhancement in the portovenous phase (**F**). 3D-MRCP (**G**) shows a cyst in close proximity to the segmental biliary ducts. Hematoxylin and eosin (H&E) staining (**H**) shows a laminated hyaline, acellular layer (*black arrows*) and the fibro-inflammatory wall of the pseudocyst (*red asterisks*) surrounded by normal liver parenchyma (*yellow asterisk*); original magnification ×400.

CL is the initial stage in the hydatid cyst development and presents as a unilocular cystic lesion without a well-defined wall, indistinguishable from a biliary cyst in imaging (Figure 1). In the absence of a focal lesion clearly compatible with CE, no diagnosis should be attempted based on clinical suspicion alone [32].

#### 3.1.2. CE1 Stage

**Figure 2:**
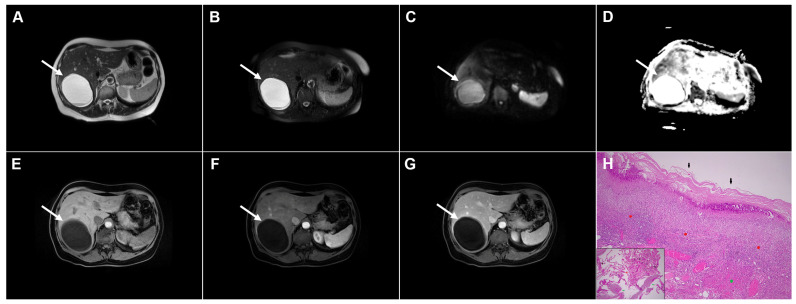
Images of a 45-year-old woman with cystic echinococcosis, WHO stage CE1. Axial T2-weighted (**A**) and T2-weighted fat-suppressed (**B**) images demonstrate a well-defined cystic lesion in liver segment VII, with a double-line sign visible along the posterior aspect (*arrow*). Diffusion-weighted image (**C**) and the corresponding ADC map (**D**) show no evidence of restricted diffusion within the cyst (*arrow*). In the precontrast T1-weighted fat-suppressed image (**E**), the cyst content (*arrow*) appears hypointense. No enhancement of the cyst wall (*arrow*) can be observed in the arterial (**F**) or portovenous (**G**) phases. Hematoxylin and eosin (H&E) staining (**H**) shows the laminar eosinophilic membranes (*black arrows*), fibro-inflammatory pseudocyst wall (*red asterisks*), and adjacent liver parenchyma (*green asterisk*); original magnification ×400. Inset shows a higher-magnification view highlighting the laminar eosinophilic membranes (*green arrows*), numerous calcified parasitic elements (*black arrows*), and the preserved, recognizable scolex (*red arrow*); original magnification ×1000.

The CE1 stage presents as a cystic lesion with a layered cyst wall, known as the double-line sign [32]. Ultrasound is usually sufficient for correct diagnosis, showing a double-line wall and hydatid sand, which can be seen as the “snowflake” sign [33]. If an MRI is performed, the characteristic appearance of the wall will clearly be detected (Figure 2) [34]. In diffusion-weighted imaging (DWI) with apparent diffusion coefficient measurements (ADC), CE1 forms were shown to have lower ADC values in comparison to simple biliary cysts [35]. This can be explained by the content of the hydatid cyst with protoscolices and other large molecules in the cystic fluid [35]. While early reports suggested lower ADC values for CE1, more recent large-scale studies have found no statistically significant difference [36].

#### 3.1.3. CE2 Stage

**Figure 3:**
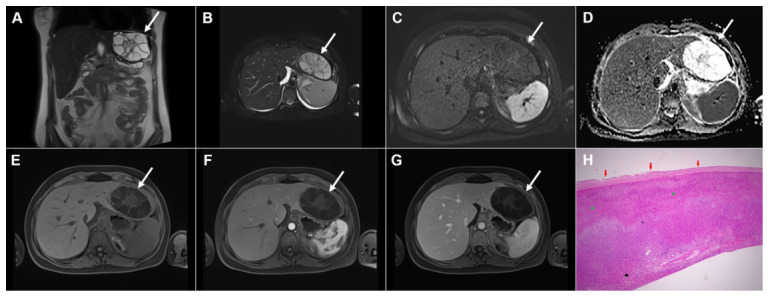
Cystic echinococcosis, WHO stage CE2, in a 25-year-old woman. Coronal T2-weighted (**A**) and axial T2-weighted fat-suppressed (**B**) images demonstrate a multilocular cystic lesion (*arrow*) in the left lateral liver segments, with daughter cysts entirely occupying the mother cyst. Diffusion-weighted image (**C**) and the corresponding ADC map (**D**) show no restricted diffusion within the lesion (*arrow*). In the precontrast T1-weighted image (**E**), the walls of the daughter cysts display an intermediate signal intensity, while the cyst fluid is hypointense (*arrow*). No enhancement of the cyst (*arrow*) was observed in arterial (**F**) or portovenous (**G**) phases. Hematoxylin and eosin (H&E) staining (**H**) shows laminated, amorphous membranes (*red arrows*), the fibro-inflammatory wall with areas of necrosis (*green asterisks*), and liver parenchyma (*black asterisk*); original magnification ×400.

CE2 typically presents as multivesicular lesions with multiple internal septations [37]. At this stage, numerous daughter cysts develop within the mother cyst, creating the characteristic “cyst-in-cyst” appearance [17]. This pattern is also referred to as the “rosette” sign or “honeycomb” pattern [17]. In T2-weighted MRI images, CE2 cysts demonstrate a uniformly high signal intensity, whereas in T1-weighted images, daughter cysts may appear hypointense relative to the mother cyst, due to the presence of free-floating protoscolices in the latter [10] (Figure 3). The cyst wall is uniformly thin and hypointense in both T1- and T2-weighted images, and the internal septations, representing the walls of the daughter cysts, are similarly hypointense [10,17]. Radiologists should keep in mind that other multilocular cystic liver lesions, particularly mucinous cystic neoplasms, can mimic the CE2 stage, and careful evaluation of internal structure is essential for accurate differentiation [37].

#### 3.1.4. CE3a and CE3b Stages

**Figure 4:**
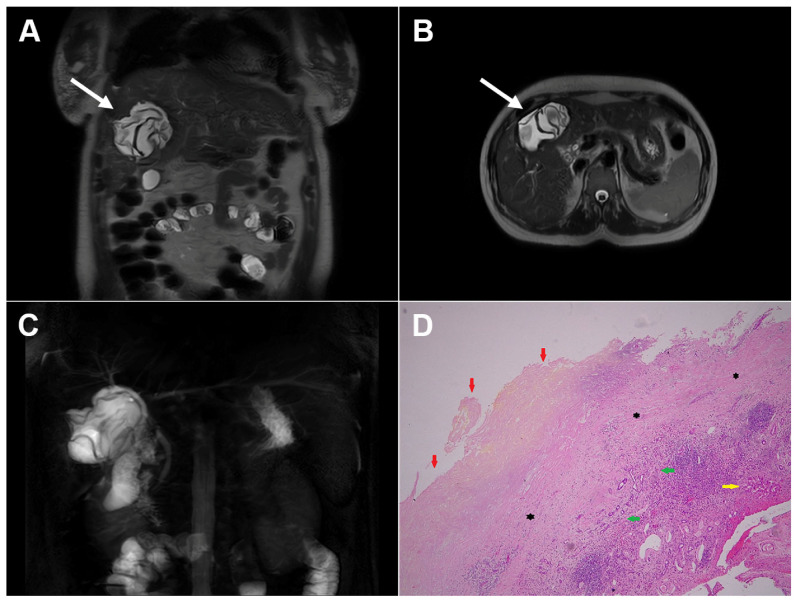
Cystic echinococcosis, WHO stage CE3a, in a 22-year-old woman. In the coronal T2-weighted image (**A**) and the axial T2-weighted image (**B**), a detached germinal membrane can be seen as a serpentine structure inside the cyst (*arrow*). The 3D-MRCP image (**C**) nicely depicts the wavy appearance of the detached germinal membrane. Hematoxylin and eosin (H&E) staining (**D**) shows the sclerotic wall of the pseudocyst (*black asterisks*), necrotic debris on the luminal surface of the pseudocyst (*red arrows*), ductular hyperplasia (*green arrows*), and a small focus of liver parenchyma (*yellow arrow*); original magnification ×400.

If the degeneration process has started, a detached germinal membrane can be seen floating in the cyst in CE stage 3a [38]. In MRI, the detached membrane is seen as a wavy T2-weighted hypointense line usually in the middle of the cyst [38]. Complete detachment of the germinal membrane results in the characteristic “water-lily” sign in imaging (Figure 4) [17].

**Figure 5:**
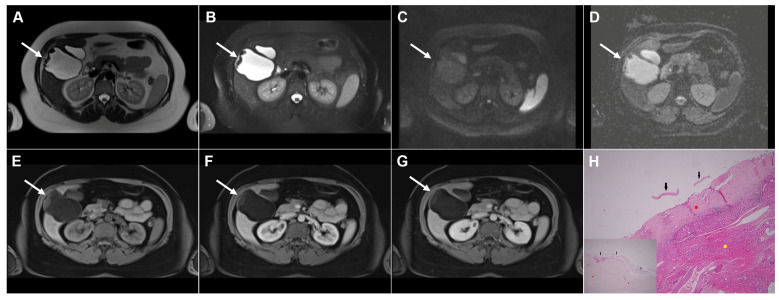
Cystic echinococcosis, WHO stage CE3a, in a 47-year-old woman. Axial T2-weighted image (**A**) and T2-weighted fat-suppressed image (**B**) demonstrate a cystic lesion in liver segment V, with a hypointense, wavy, detached germinal membrane adherent to the lateral wall of the cystic lesion (*arrow*). Diffusion-weighted image (**C**) and the corresponding ADC map (**D**) show no restricted diffusion (*arrow*). In the T1-weighted fat-suppressed image (**E**), the detached membrane exhibits a slightly higher signal intensity, with no enhancement in arterial (**F**) or portovenous (**G**) phases (*arrow*). Hematoxylin and eosin (H&E) staining (**H**) shows the laminar eosinophilic membranes (*black arrows*), fibro-inflammatory pseudocyst wall (*red asterisk*), and adjacent liver parenchyma (*yellow asterisk*); original magnification ×400. Inset shows a higher-magnification view highlighting calcified and dystrophic structures consistent with degenerative parasitic forms (*black arrows*) and laminar eosinophilic membranes (*red asterisks*); original magnification ×1000.

It is important to note that detached membranes might have very different appearances from the typically described ones, thus occasionally causing diagnostic dilemmas [37]. In this regard, the detached membrane is not always seen floating inside the cyst but may appear adjacent to the cyst wall (Figure 5).

**Figure 6:**
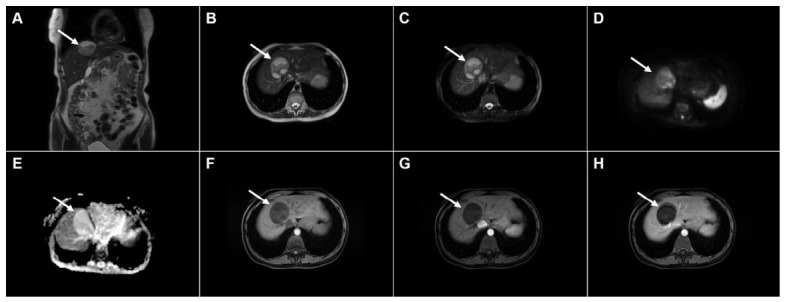
Images of a 77-year-old woman with cystic echinococcosis, WHO stage CE3b. Coronal T2-weighted image (**A**) demonstrates a heterogeneous cystic lesion (*arrow*) in liver segment VIII. Axial T2-weighted (**B**) and axial T2-weighted fat-suppressed (**C**) images show a few daughter cysts embedded within the hydatid matrix (*arrow*). The lesion (*arrow*) demonstrates no restricted diffusion in the diffusion-weighted image (**D**) and the corresponding ADC map (**E**). In the precontrast T1-weighted fat-suppressed image (**F**), the hydatid matrix exhibits a slightly higher signal intensity compared to the daughter cysts (*arrow*). No enhancement of the lesion (*arrow*) can be observed in arterial (**G**) or portovenous (**H**) phases.

Further degeneration of the cyst leads to the CE3b stage, where daughter cysts are embedded in the hydatid matrix made of the degenerated germinal layer, broken daughter vesicles, and hydatid sand [38]. In this stage, T2-weighted images might display a characteristic wheel/spoke pattern consisting of the dense hydatid matrix separating different numbers of locules inside representing daughter cysts (Figure 6) [38].

#### 3.1.5. Inactive Stages (CE4 and CE5)

**Figure 7:**
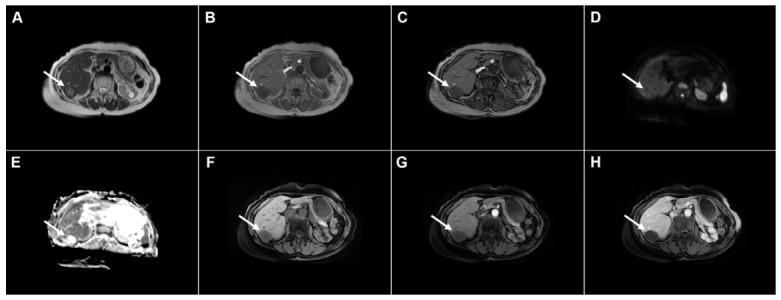
Cystic echinococcosis, WHO stage CE4, in a 68-year-old woman. Axial T2-weighted image (**A**) demonstrates a heterogeneous, semi-solid lesion (*arrow*) located posteriorly in liver segment VI. In-phase (**B**) and out-of-phase (**C**) images show that the lesion (*arrow*) is hypointense. No restricted diffusion can be observed (*arrow*) in the diffusion-weighted image (**D**) or in the corresponding ADC map (**E**). T1-weighted fat-suppressed images obtained before (**F**) and during the arterial (**G**) and portovenous phase (**H**) show no post-contrast enhancement, consistent with an avascular lesion (*arrow*).

The inactive stages are considered to be CE4 and CE5, in which the cyst loses its typical daughter cysts and internal septations and presents as a pseudo-solid lesion with solidified or degenerating content [17]. In T2-weighted MRI, the cyst may show a low-to-intermediate signal intensity, reflecting the degenerative material, with no clearly identifiable daughter cysts (Figure 7) [17]. The pericyst remains visible as a thin hypointense rim [17].

**Figure 8:**
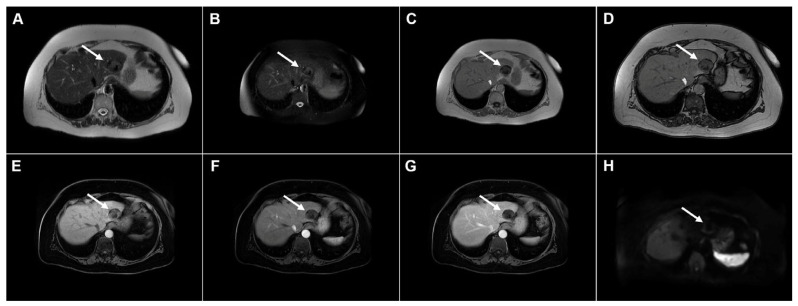
Cystic echinococcosis, WHO stage CE5, in a 72-year-old man. Axial T2-weighted (**A**) and T2-weighted fat-suppressed (**B**) images demonstrate a heterogeneous, predominantly hypointense, solid-appearing lesion (*arrow*) in liver segment II. In-phase (**C**) and out-of-phase (**D**) images show the lesion (*arrow*) as hypointense, with a signal void along the cyst wall consistent with calcifications. In the T1-weighted fat-suppressed image (**E**), the lesion is heterogeneously hypointense, with a linear hypointense rim along the cyst wall (*arrow*), compatible with calcifications. No enhancement can be observed in the arterial (**F**) or portovenous phase (**G**) (*arrow*). In diffusion-weighted imaging (**H**), the lesion is isointense relative to the surrounding liver parenchyma (*arrow*).

In CE4, calcification in the cyst wall occurs, while in CE5 the cyst wall is typically thickened and heavily calcified [38]. Although MRI can only indirectly suggest the presence of calcifications based on the low signal intensity in out-of-phase images, susceptibility-weighted imaging and other susceptibility-sensitive sequences may increase sensitivity for detecting mineralized components (Figure 8).

**Figure 9:**
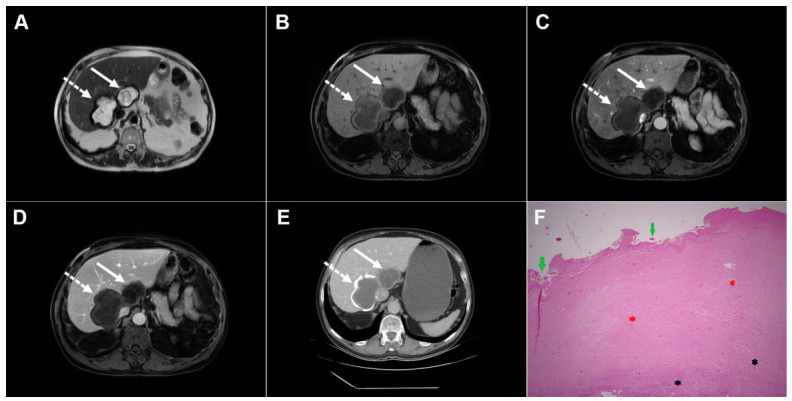
Images of a 75-year-old man with two cystic echinococcal lesions, WHO stage CE3b. Axial T2-weighted image (**A**) demonstrates two cystic lesions in the caudate lobe (*arrow*) and liver segment VII (*dotted arrow*). T1-weighted image (**B**) shows the heterogeneously low signal intensity of the cystic fluid, with no enhancement in arterial (**C**) or portovenous (**D**) phase images (*arrows and dotted arrow*). CT scan (**E**) in the same patient reveals coarse calcifications in the wall of the lesion in segment VII (*dotted arrow*), whereas the lesion in the caudate lobe is not calcified (*arrow*). Hematoxylin and eosin (H&E) staining (**F**) shows a laminated hyaline acellular layer (*green arrows*), necrotic debris (*red asterisks*), and a fibro-inflammatory wall of the pseudocyst (*black asterisks*); original magnification ×400.

In difficult cases, both CT and MRI might be performed in order to achieve a correct diagnosis [39]. Radiologists and clinicians should bear in mind that even though wall calcifications are typically present in CE4 and CE5, they can also occur in active and transitional cases; thus, they do not necessarily indicate parasite inactivity (Figure 9) [40]. Therefore, in cases of CE with a calcified wall, further ultrasound or MRI evaluation of the cyst content is suggested for assessment of activity. Due to the solid appearance in T2-weighted images, CE4 and CE5 may resemble other solid focal liver lesions [37]. Besides a hypointense wall, which can suggest calcifications, a crucial feature for differentiating CE lesions from other solid hepatic lesions is a lack of post-contrast opacification [37].

### 3.2. Complications of Cystic Echinococcosis

Complications of cystic echinococcosis are relatively rare, as most cysts remain asymptomatic for a long period of time [14]. The most common complications are mechanical, resulting from cyst rupture or compression of biliovascular structures [10]. Less frequently, CE may lead to secondary infection or anaphylactic reactions.

**Figure 10:**
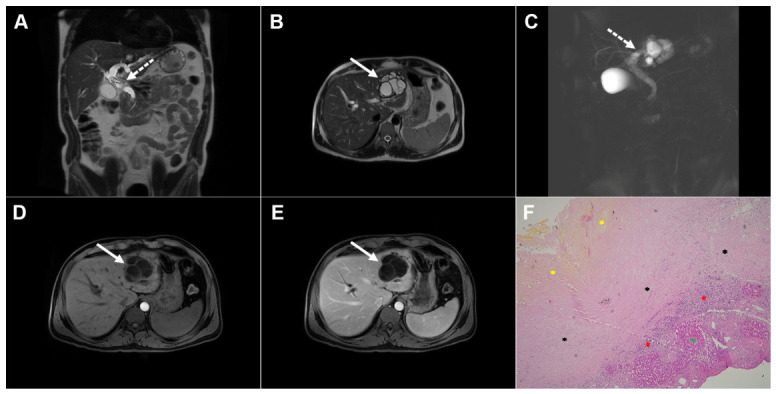
Images of a 25-year-old man with complicated cystic echinococcosis. Coronal T2-weighted image (**A**) demonstrates a clearly visible germinal membrane within the common bile duct (*dotted arrow*), consistent with intrabiliary rupture of the cyst. Axial T2-weighted image (**B**) shows a multilocular cystic lesion (*arrow*) in liver segment II. 3D magnetic resonance cholangiopancreatography (MRCP) (**C**) reveals a large echinococcal cyst communicating with the bile duct (*dotted arrow* ). T1-weighted image (**D**) demonstrates the low signal intensity of the cyst fluid and the intermediate signal intensity of the daughter cyst walls (*arrow*). No enhancement can be observed in the portovenous phase image (*arrow*) (**E**). Hematoxylin and eosin (H&E) staining (**F**) shows a fibroinflammatory wall of the pseudocyst (*black asterisks*), necrotic debris on the inner surface of the pseudocyst (*yellow asterisks*), proliferated bile ducts (*red asterisks*), and surrounding liver parenchyma (*green asterisk*); original magnification ×400.

Rupture of the echinococcal cyst most commonly occurs in the biliary tree due to increased intracystic pressure, which compresses adjacent bile ducts and may cause necrosis of the cyst wall [16]. In MRI, a ruptured cyst may show a defect in the cyst wall and continuity with an adjacent bile duct, although this characteristic sign is infrequently seen. Furthermore, a beak-like projection extending from the cyst wall towards the bile duct might be seen. Detached germinal membrane fragments may appear as T2-weighted hypointense linear structures within the biliary ducts (Figure 10). Biliary obstruction from hydatid sand can lead to upstream ductal dilatation and may be complicated by cholangitis, sometimes visible as enhancement of duct walls on contrast-enhanced sequences. In contrast to previously described findings in cases where intrabiliary rupture is obvious in MRI, in many patients, the communication of the cyst with small bile ducts through minute fissures remains occult [41].

**Figure 11:**
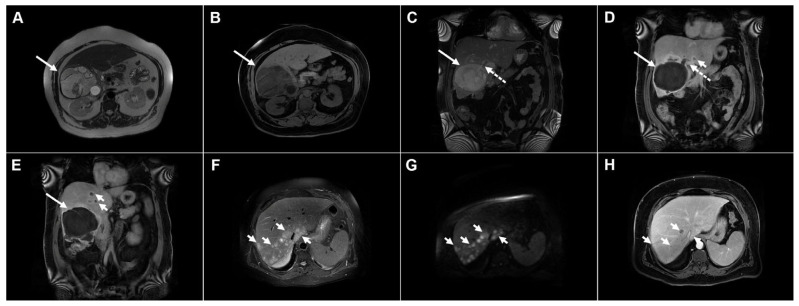
Images of a 56-year-old man with an infected echinococcal cyst. Axial T2-weighted image (**A**) demonstrates a heterogeneous cystic lesion (*arrow*) with a few daughter cysts embedded in the hydatid matrix in liver segment VI. T1-weighted fat-suppressed image (**B**) shows the low signal intensity of the daughter cysts and the intermediate signal intensity of the hydatid sand (*arrow*). Coronal T2-weighted (**C**) and coronal post-contrast images (**D**,**E**) demonstrate a well-defined cystic lesion (*arrow*) communicating with the bile duct (*dotted arrow*). Multiple hepatic abscesses can also be observed in the surrounding liver parenchyma (*arrowheads*). Axial T2-weighted (**F**), diffusion-weighted (**G**), and portovenous phase (**H**) images show multiple small hepatic abscesses grouped in liver segment VII (*arrowheads*).

Secondary infection occurs when bacteria contaminate hydatid fluid via communication with the biliary tree [42]. Infected echinococcal cysts may present with a thickened, irregularly enhancing cyst wall, pericystic edema, and hyperperfusion of the adjacent liver parenchyma. Small abscesses may also form around the cyst (Figure 11) [10]. Clinical signs such as fever, abdominal pain, and leukocytosis in a patient with a known hydatid cyst should raise suspicion for superinfection [20].

**Figure 12:**
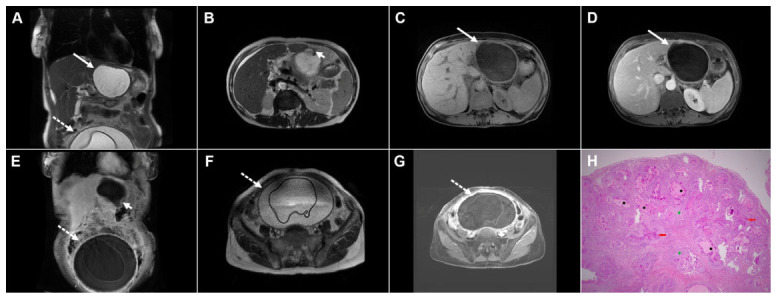
Intraperitoneal rupture and disseminated echinococcosis in a 72-year-old woman. Coronal T2-weighted image (**A**) demonstrates cystic echinococcosis, WHO stage CE3a, in the left liver lobe (*arrow*), while a pelvic hydatid cyst is partially depicted in the lower abdomen (*dotted arrow*). Axial T2-weighted image (**B**) shows the site of cyst rupture (*arrowhead*). Axial T1-weighted fat-suppressed image (**C**) demonstrates a heterogeneous content within the cyst (*arrow*), without enhancement of the portovenous phase image (**D**) (*arrow*). Post-contrast coronal image (**E**) depicts the ruptured liver hydatid cyst (*arrowhead*) and the large pelvic hydatid cyst (*dotted arrow*). Axial T2-weighted (**F**) and axial T1-weighted post-contrast (**G**) images through the pelvis show the pelvic hydatid cyst with a wavy, detached germinal membrane (*dotted arrow*). Hematoxylin and eosin (H&E) staining (**H**) shows pelvic hydatid cysts with laminated, amorphous membranes and occasional degenerated scolices (*black asterisks*), a fibro-inflammatory wall surrounding the laminated membrane (*green asterisks*), and numerous foreign-body-type giant cells (*red arrows*); original magnification ×400.

Intraperitoneal rupture is more likely if the cyst is located peripherally beneath the anterior or inferior surface of the liver or in the left liver lobe [43]. Rupture may occur spontaneously or after abdominal trauma. Severe abdominal pain accompanied by allergic reactions may be the initial presentation, with potential progression to disseminated peritoneal hydatidosis [44]. Initially, MRI may demonstrate free fluid with cystic content in the peritoneal cavity. Furthermore, a defect in the wall of the hydatid cyst or a change in morphology might be seen [20]. As a consequence of hydatid cyst intraperitoneal rupture, peritoneal hydatidosis might occur with multiple intraperitoneal hydatid cysts [45]. Besides disseminated peritoneal disease, isolated pelvic hydatid cysts might be seen in 2.2% of patients (Figure 12) [46]. Pelvic hydatid cysts may mimic adnexal tumors in female patients [46]. However, when a hepatic hydatid cyst is present, secondary pelvic involvement should be considered. Additionally, if other signs of echinococcal cysts are present, such as detached membranes, or daughter cysts, these features favor the diagnosis.

**Figure 13:**
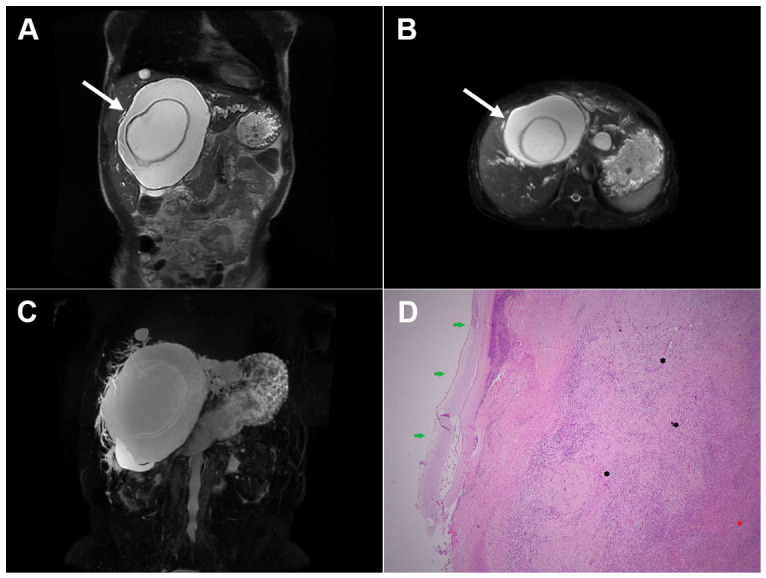
Biliary obstruction due to cystic echinococcosis, WHO stage CE3a, in a 69-year-old man. Coronal T2-weighted (**A**) and axial T2-weighted fat-suppressed (**B**) images demonstrate a large cystic lesion (*arrow*) in liver segments IV and VIII containing a wavy, detached germinal membrane. MRCP (**C**) demonstrates intrahepatic biliary dilatation secondary to extrinsic compression by the cyst wall. Hematoxylin and eosin (H&E) staining (**D**) highlights the inner surface of the pseudocyst, which is lined by acellular, lamellar, eosinophilic, hyalinized material consistent with a laminated (cuticular) membrane, a feature compatible with a hydatid cyst (*green arrows*); the fibro-inflammatory cyst wall (*black asterisks*) and the adjacent liver parenchyma (*red asterisks*) are also shown; original magnification ×400.

Compression of large biliary ducts as a complication of CE may result in biliary dilatation, while compression of hepatic veins can lead to Budd–Chiari syndrome (Figure 13) [10].

**Figure 14:**
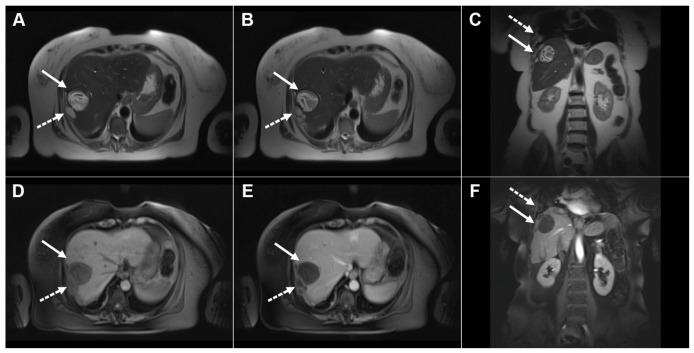
Complicated hydatid cyst in a 73-year-old female. Axial T2-weighted images (**A**,**B**) demonstrate a cystic lesion (*arrows*) with a detached germinal membrane and a small rupture, resulting in extrahepatic extension (*dotted arrows*). Coronal T2-weighted image (**C**) shows transdiaphragmatic extension of the cyst into the thoracic cavity (*arrow*, *dotted arrow*). The liver lesion (*arrow*) and extrahepatic part (*dotted arrow*) are hypointense in the T1-weighted fat-suppressed image (**D**), without enhancement in the portovenous phase image (**E**). Coronal post-contrast image (**F**) demonstrates absent enhancement of both the liver lesion (*arrow*) and the transdiaphragmatic cystic extension (*dotted arrow*).

Another rare complication of hepatic hydatid cysts is exophytic cyst growth through the liver’s bare area, allowing transdiaphragmatic migration, which can be visualized as a cystic lesion extending into the lungs or mediastinum (Figure 14) [47].

## 4. Imaging Findings of Alveolar Echinococcosis

### 4.1. MRI Features of Alveolar Echinococcosis

**Figure 15:**
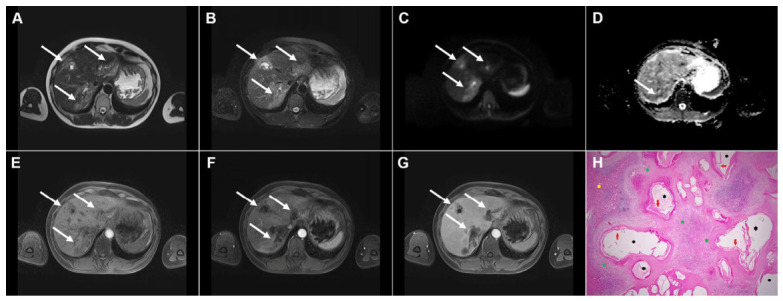
Alveolar echinococcosis in a 57-year-old woman. Multiple irregularly shaped, heterogeneously hyperintense lesions (*arrows*) can be seen in liver segments II, VIII, and VII in T2-weighted (**A**) and T2-weighted fat-suppressed images (**B**), with small eccentric necrotic areas. In axial diffusion-weighted imaging (**C**) and the corresponding ADC map (**D**), the lesions show restricted diffusion. In a native T1-weighted fat-suppressed image (**E**), the lesions (*arrows*) are hypointense, with only discrete peripheral enhancement in the arterial (**F**) and portovenous phase (**G**) images. Hematoxylin and eosin (H&E) staining (**H**) shows multilocular pseudocystic formations (*black asterisks*) with walls composed of a fibro-inflammatory zone (*green asterisks*), necrotic debris, and irregular hydatid membranes inside the pseudocysts (*red arrows*), as well as surrounding liver parenchyma (*yellow asterisk*); original magnification ×400.

AE is a considerably rarer disease in comparison to CE [1]. The rarity of the disease, together with non-specific radiological appearance, leads in many cases to misdiagnosis [48,49]. The fact that AE, if untreated, is a fatal disease in more than 90% of patients, indicates the necessity of considering this entity, especially when epidemiological data are present [50]. Concerning the diagnosis of CE, ultrasound is the cornerstone of diagnosis, staging, and follow-up in most typical cases, while CT/MRI is often useful in atypical or complicated lesions. On the other hand, in AE, ultrasound is not sufficient for diagnosis and further MR imaging is advised. MRI is a highly significant complementary modality, particularly for lesion characterization, biliary involvement, and staging context [51,52]. The most characteristic finding that can raise suspicion of AE is a multicystic, ill-defined infiltrative lesion consisting of multiple small vesicles inside or at the borders of the solid component (Figure 15) [52]. Multivesicular structure is considered to be a pathognomonic finding for AE and is best visualized in T2-weighted images [53]. Vesicles are usually less than 1 cm and can be seen embedded in a solid component that consists of granulomatous tissue [51,52,53]. Solid tissue typically has a low signal intensity in T2-weighted images and a low-to-intermediate signal intensity in T1-weighted images [52].

**Figure 16:**
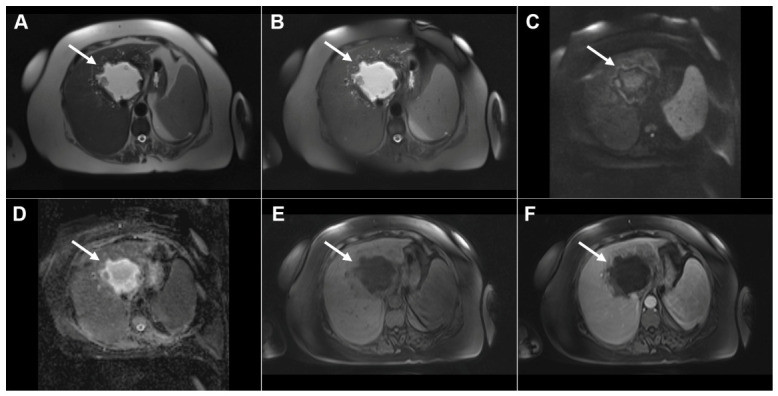
Alveolar echinococcosis in a 73-year-old woman. Axial T2-weighted image (**A**) demonstrates a necrotic lesion (*arrow*) centrally located in liver segments IV and VIII. In the T2-weighted fat-suppressed image (**B**), multiple tiny hyperintense vesicles (*arrow*) can be observed surrounding the central necrotic area. Diffusion-weighted image (**C**) and the corresponding ADC map (**D**) show slight restricted diffusion in the solid portion of the lesion (*arrow*). The lesion (*arrow*) is hypointense in the T1-weighted fat-suppressed image (**E**), with mild enhancement of the peripheral solid component in the portovenous phase image (**F**), representing the host granulomatous reaction. As serologic testing demonstrated high positivity for *Echinococcus multilocularis*, percutaneous biopsy was not performed.

Areas of liquefactive necrosis occur in larger lesions and mostly exhibit a high T2-weighted signal intensity and a low-to-intermediate signal intensity in T1-weighted images (Figure 16) [52]. Although the mass does not enhance, the surrounding fibroinflammatory component might show slight enhancement in the delayed phase [53].

If CT is performed, an infiltrating tumor-like hepatic mass with scattered linear or plaquelike calcifications can be seen [52]. In some cases, AE can appear as predominantly cystic lesions. Minimal calcifications, limited infiltrative features, or unusual locations can make differentiation from other liver lesions difficult [49]. These atypical forms highlight the need for careful radiologic assessment, including evaluation of lesion margins, internal architecture, and enhancement patterns.

Becce et al. [54] have shown that diffusion-weighted imaging with ADC measurement might provide additional features for the differentiation of AE and malignant mimickers. The low ADC values in AE reflect the high cellularity and viscosity of the solid, fibroinflammatory component. However, in AE cases, ADC values are higher in contrast to malignant liver lesions such as cholangiocarcinoma [54,55]. Nevertheless, there is no determined cut-off ADC value for discrimination among these lesions [54,55].

### 4.2. Kodami Classification

Kodama et al. [53] have proposed a classification of imaging findings for AE, which includes five types. In type 1, only multiple aggregated small cysts are visualized. Types 2 and 3 are the most common types, where multiple small cysts within a solid component can be seen. Besides the solid component, in type 3, irregular central necrotic part can also be observed. These two types, together with type 1, present active forms of AE. Type 4, where only a solid component without cysts can be seen, and type 5, presenting as a large cyst without a solid component, are inactive and rarely seen AE lesions.

Taking into account the metastatic potential of AE, the WHO-IWGE has classified AE into different PNM stages (parasite lesion, neighboring organs, and metastases), which influence further clinical decisions and treatment [56]. Category P is used for the description of parasitic dissemination in the liver, N determines the involvement of adjacent organs, and M refers to distant metastases [56].

### 4.3. Complications

Complications of AE are common due to its infiltrative nature and occur more frequently than in CE, affecting the clinical course and prognosis significantly [10]. The most frequent complications include biliary involvement, vascular infiltration, and extrahepatic spread [20].

**Figure 17:**
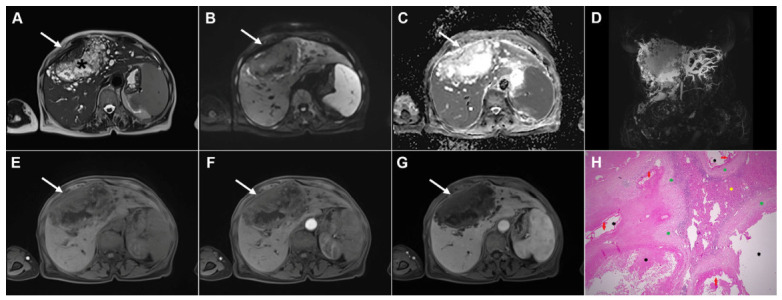
Alveolar echinococcosis in a 77-year-old woman. Axial T2-weighted image (**A**) demonstrates a large heterogeneous lesion (*arrow*) predominantly located in liver segments IV and VIII, with an irregular central necrotic area (*asterisk*) surrounded by multiple hyperintense vesicles. Diffusion-weighted image (**B**) and the corresponding ADC map (**C**) show only slightly restricted diffusion at the periphery of the lesion (*arrow*). 3D MRCP (**D**) demonstrates the necrotic portion of the lesion and biliary dilatation in the surrounding parenchyma due to mass effect. The lesion (*arrow*) is hypointense in T1-weighted fat-suppressed imaging (**E**) and avascular in both arterial (**F**) and portovenous phase (**G**) images. Hematoxylin and eosin (H&E) staining (**H**) shows multilocular pseudocystic formations (*black asterisks*) with walls composed of a fibro-inflammatory zone (*green asterisks*), necrotic debris, and irregular hydatid membranes inside the pseudocysts (*red arrows*), as well as surrounding liver parenchyma (*yellow asterisk*); original magnification ×400.

Biliary involvement represents the leading complication of AE with an incidence of 10% to 30% [57]. Unlike CE, where rupture into the biliary tree is the main mechanism, in AE, biliary complications arise from direct infiltration or compression of bile ducts by the parasitic mass. Patients typically present with cholangitis or jaundice [58]. Biliary obstruction is visualized in MRI as upstream ductal dilatation with abrupt termination at the level of the lesion (Figure 17). Cholangitis may be evident as thickening and enhancement of the bile duct walls in post-contrast sequences.

**Figure 18:**
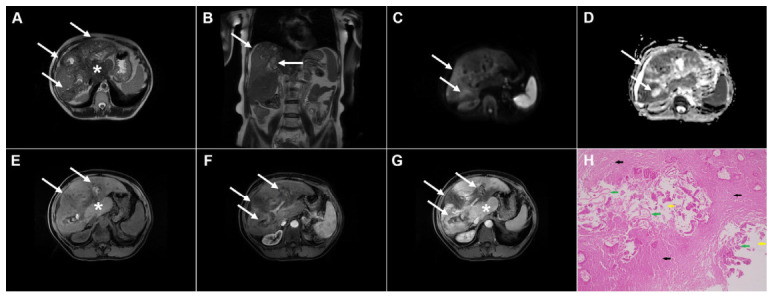
Images of a 56-year-old woman with pseudocirrhotic liver configuration resulting from long-standing alveolar echinococcosis. Axial (**A**) and coronal (**B**) T2-weighted images demonstrate multiple irregular, infiltrative lesions that are heterogeneously slightly hyperintense, with areas of necrosis (*arrows*). The lesions (*arrows*) show mild hyperintensity in the diffusion-weighted image (**C**) without significant restricted diffusion on the ADC map (**D**). In the T1-weighted fat-suppressed image (**E**), the lesions are hypointense, with foci of high signal intensity in necrotic areas (*arrows*). In arterial (**F**) and portovenous phase (**G**) images, the lesions are predominantly avascular, with slight enhancement noted in the lesion located in liver segment VII (*arrows*). Caudate lobe hypertrophy is also present (*asterisk* on **A**,**E**,**G**). Hematoxylin and eosin (H&E) staining (**H**) shows amorphous and partially granular necrotic tissue material (*black arrows*) and numerous microcysts (*yellow arrows*) lined by hyaline membranous structures (*green arrows*); original magnification ×400.

Vascular involvement is a serious complication that occurs due to the infiltrative growth pattern of AE [58]. The parasite can invade portal vein branches, hepatic veins, and, in advanced cases, the inferior vena cava [59]. In longstanding disease, infiltration of hilar structures and vascular involvement can lead to the development of portal hypertension and pseudocirrhosis (Figure 18) [59]. The liver may show segmental or lobar atrophy in areas affected by vascular compromise, while compensatory hypertrophy may be observed in unaffected segments, creating the appearance of pseudocirrhosis [60,61].

Distant metastases, though rare, can occur and most commonly affect the lungs, brain, and bones [62]. These metastatic lesions maintain the characteristic multivesicular appearance of the primary lesion.

Although less common than in CE, secondary bacterial infection of AE lesions can occur, particularly when there is communication with the biliary system [63]. Infected lesions demonstrate thickened, irregularly enhancing walls, increased surrounding edema, and possible gas formation within necrotic areas [63].

## 5. Differential Diagnosis of Cystic Echinococcosis

### 5.1. Biliary Cysts

**Figure 19:**
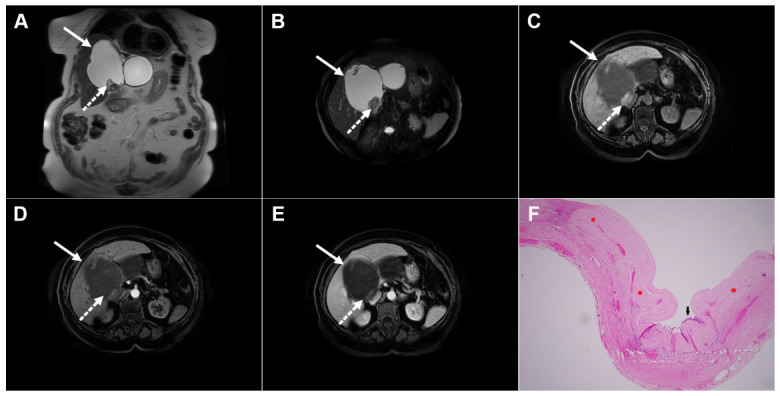
Images of a 73-year-old woman with a complicated biliary cyst. Coronal T2-weighted image (**A**) and axial T2-weighted fat-suppressed image (**B**) demonstrate a bilocular cystic lesion (*arrow*) involving liver segments VIII and V and the caudate lobe, with a heterogeneously hypointense content adjacent to the posterior wall (*dotted arrow*), representing retracted coagulum. Axial T1-weighted fat-suppressed image (**C**) shows hypointense cystic fluid (*arrow*), in contrast to the high-signal coagulum (*dotted arrow*). No enhancement can be observed in arterial (**D**) or portovenous phase (**E**) images (*arrows and dotted arrow*s). Hematoxylin and eosin (H&E) staining (**F**) shows biliary epithelium with features of low-grade epithelial dysplasia (*black arrow*) and the sclerohyalinized cyst wall (*red asterisks*); original magnification ×400.

**Figure 20:**
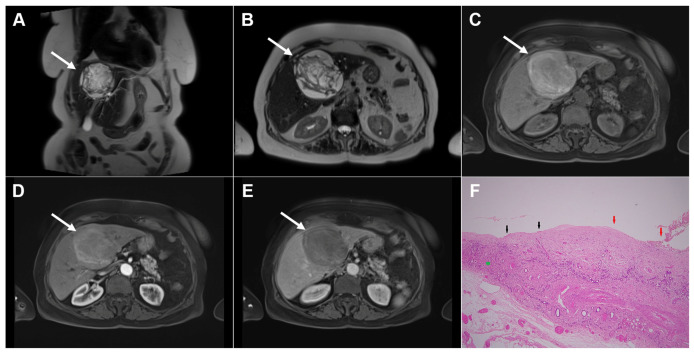
Complicated biliary cyst in a 53-year-old woman. Coronal T2-weighted (**A**) and axial T2-weighted (**B**) images demonstrate a cystic lesion (*arrow*) with multiple hypointense bands within the lesion, representing retracted coagulum. In the T1-weighted fat-suppressed image (**C**), the cystic content (*arrow*) is homogeneously hyperintense, consistent with hemorrhage. No enhancement can be observed in arterial (**D**) or portovenous (**E**) phases images (*arrow*). Hematoxylin and eosin (H&E) staining (**F**) shows small foci of preserved single-layer cuboidal epithelium without features of atypia or dysplasia (*black arrows*), the ulcerated and denuded inner surface of the cyst (*red arrows*), and the liver parenchyma (*green asterisk*); original magnification ×400.

Biliary cysts are the most common benign liver lesions, with a reported prevalence ranging from 2.5% to 18% [64]. These congenital lesions arise from aberrant bile ducts that do not communicate with the main biliary tree [65]. The radiological diagnosis of simple biliary cysts is usually unequivocal. They appear as well-defined, unilocular lesions that are hypointense in T1-weighted images and homogeneously hyperintense in T2-weighted images, with a very thin wall [66]. The first stage of CE, as defined by WHO-IWGE, which presents as a cystic lesion, is indistinguishable from a simple biliary cyst [32]. In these cases, only positive serology tests might raise the suspicion of CE. Lesion morphology may provide additional information in differential diagnosis. While biliary cysts generally present with a regular, spherical morphology, cystic echinococcosis, manifesting as a CL lesion, may present with an irregular shape, a feature that, when correlated with relevant epidemiological data, may suggest an infectious etiology. Concerning the differential diagnosis of these two entities, Inan et al. have shown that DWI with high b values together with ADC measurements may facilitate distinction [35]. These authors have shown that the mean ADC of hydatid cysts was significantly lower than the ADC values of simple cysts [35]. In contrast to CL, CE1-stage and CE2-stage lesions are usually not mistaken for simple biliary cysts due to their characteristic appearance [32]. Nevertheless, complications such as internal hemorrhage can alter the imaging features of biliary cysts, occasionally leading to misdiagnosis with other complex cystic liver lesions, including CE [66]. Retraction of the coagulum may produce a T2-weighted signal pattern that closely mimics the appearance of a detached germinative membrane, characteristic of CE3a-stage lesions (Figure 19 and Figure 20) [67,68]. Moreover, the absence of post-contrast enhancement of internal septa or membranes is seen in both complicated biliary cysts and CE [66]. In these cases, the T1-weighted signal intensity of the cyst content becomes crucial for differentiation. Hemorrhagic biliary cysts typically show internal T1-weighted hyperintensity, while the content of echinococcal cysts is usually hypointense in T1-weighted images [67,68].

**Figure 21:**
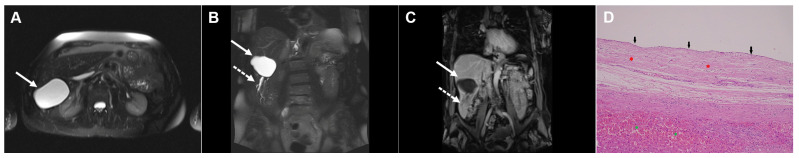
Images of a 28-year-old woman with a biliary cyst. Axial T2-weighted image (**A**) demonstrates a simple cystic lesion (*arrow*) in liver segment VI. Coronal T2-weighted (**B**) and post-contrast images (**C**) show biliary dilatation extending caudally (*dotted arrows*) from the lesion (*arrows*). Hematoxylin and eosin (H&E) staining (**D**) shows cuboidal, predominantly flattened epithelium without features of atypia or dysplasia (*black arrows*), the fibrous wall of the cyst (*red asterisks*), and the surrounding liver parenchyma (*green asterisks*); original magnification ×400.

Although rare, large biliary cysts may cause extrinsic compression of the bile ducts, which can mimic obstructive features seen in CE when lesions are located perihilary (Figure 21) [69]. To prevent potential misdiagnoses, careful assessment of lesion morphology, cyst content signal characteristics, and the presence or absence of daughter cysts or detached membranes is essential to differentiate complicated biliary cysts from cystic echinococcosis, guiding appropriate management and avoiding unnecessary interventions.

### 5.2. Mucinous Cystic Neoplasm of the Liver

**Figure 22:**
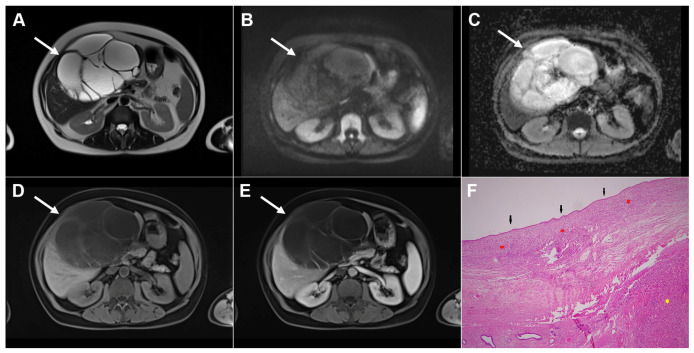
Mucinous cystic neoplasm (MCN) of the liver in a 33-year-old woman. A large, well-defined multilocular cystic lesion (*arrow*) with irregularly distributed internal septa can be seen in liver segments IV and V in the axial T2-weighted image (**A**). No diffusion restriction can be observed in DWI (**B**) or the ADC map (*arrow*) (**C**). In the axial T1-weighted fat-suppressed image (**D**), the lesion (*arrow*) is hypointense, while the septa demonstrate an intermediate signal intensity with mild enhancement (*arrow*) in the portovenous phase image (**E**). No solid component is present within the lesion. Hematoxylin and eosin (H&E) staining (**F**) shows a single-layer epithelium that is partly mucin-producing, partly cuboidal, and partly flattened, with only focal features of low-grade dysplasia (*black arrows*), the subepithelially located ovarian-type stroma (*red asterisks*), and the surrounding liver parenchyma (*yellow asterisk*); original magnification ×400.

Mucinous cystic neoplasm of the liver, previously known as biliary cystadenoma, is a rare liver tumor representing 5% of all cystic liver lesions [70]. Nowadays, it is known that these tumors originate from ectopic ovarian stroma, which migrates to the liver during the embryogenic period [71]. The presence of ovarian stroma is mandatory for pathohistological diagnosis. These tumors are exclusively seen in women, most commonly middle-aged, and for unknown reasons are usually localized in the left liver lobe, presumably segment IV [71,72]. MCN typically presents as multilocular cystic lesions with various numbers of internal septations and irregularly thickened walls which enhance in contrast-enhanced MRI (Figure 22) [72,73]. The internal fluid content has a variable appearance in T1-weighted images, ranging from T1-weighted hypointensity to hyperintensity due to its being protein-rich [72].

**Figure 23:**
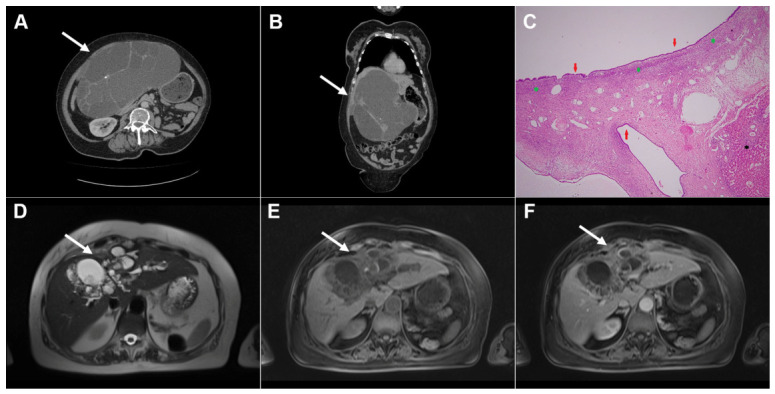
Images of a 57-year-old woman with recurrent mucinous cystic neoplasm (MCN) of the liver. Axial (**A**) and coronal (**B**) CT images demonstrate a large multilocular lesion (*arrow*) with irregularly thickened internal septations and a minor solid component. Hematoxylin and eosin (H&E) staining (**C**) shows a single-layered, mucin-producing epithelium (*red arrows*), ovarian-type stroma (*green asterisk*), and surrounding liver parenchyma (*black asterisk*); original magnification ×400. Axial T2-weighted MR image (**D**) shows local recurrence (*arrow*) at the previous surgical site, appearing as an irregular cystic lesion with a solid component and associated biliary dilatation in the surrounding liver parenchyma. The lesion (*arrow*) is hypointense in the T1-weighted fat-suppressed image (**E**), with enhancement of the solid component in the portovenous phase image (**F**).

If CT is performed, in certain cases, internal septal calcifications might be noted. Rarely, a fluid–fluid level might be seen due to internal hemorrhage [72]. The presence of a solid component indicates malignant transformation, which is seen in 5–9% of patients [74]. Since both MCN and cystic echinococcosis CE2 present as multilocular cystic lesions, differential diagnosis might be difficult [75,76,77]. Taking into account that the therapeutic approach is quite different for these two entities, preoperative distinction is very important [77]. While in the case of MCN, radical excision is necessary, in CE, percutaneous treatment or a partial cystectomy might be performed. Although recurrence of MCN might occur even after total excision in 10–20% of cases, its rate is very high (80%) after partial excision, indicating the importance of correct preoperative differentiation (Figure 23) [76]. Nevertheless, there are certain imaging features that may facilitate the establishment of a correct diagnosis. The presence of irregularly thickened septations, internal nodularity or a solid enhancing component, and lesion-associated upstream biliary dilatation strongly suggests a diagnosis of MCN [73]. When solid mural nodules or papillary projections are absent, the diagnosis might be challenging. In such difficult cases, the distribution and size of the locules in different planes should be assessed in detail. The locules in MCN lack the uniform, well-defined shape and size which is characteristic of daughter cysts seen in echinococcal cysts [73]. Concerning the location in the liver, MCN usually occurs in liver segment IV, while CE is more frequently encountered in the right liver lobe due to the nature of portal blood flow [78]. If precise preoperative differentiation is not possible, MCN should be strongly considered, and multidisciplinary surgical consultation is warranted when imaging is suspicious.

**Figure 24:**
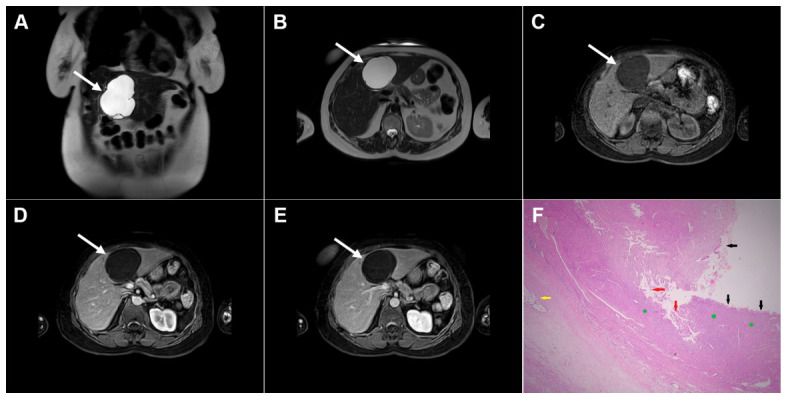
Mucinous cystic neoplasm (MCN) of the liver in a 53-year-old woman. Coronal (**A**) and axial T2-weighted (**B**) images demonstrate an oligolocular cystic lesion (*arrow*) in liver segment IV with only a few internal septations. In the T1-weighted fat-suppressed image (**C**), the lesion (*arrow*) is uniformly hypointense, with no enhancement detected in the wall and septa in arterial (**D**) or portovenous (**E**) phase images (*arrow*). Note that the septa–wall interface is smooth and that no mural nodularity can be observed, consistent with a simple cystic morphology. Hematoxylin and eosin (H&E) staining (**F**) shows a single-layer epithelium that is partly mucin-producing and partly cuboidal, with only focal features of low-grade dysplasia (*black arrows*), subepithelially located ovarian-type stroma (*green asterisks*), ulcerated cyst surface (*red arrows*), and cholesterol crystals (*yellow arrow*); original magnification ×400.

In contrast to its characteristic multilocular appearance, MCN may occasionally present as a cystic lesion with a few internal septations without detectable post-contrast opacification masquerading CE3a lesions or biliary cysts (Figure 24) [73]. A wavy or serpentine appearance of detached membrane floating inside the lesion suggests CE3a lesions. Regarding distinguishing between MCN and septated biliary cysts, Kovacs et al. [79] found that analysis of the relationship of lesion sepatations to the outer cyst wall contour leads to high diagnostic accuracy. While in biliary cysts septations always arise from external macrolobulation of the cyst wall, in MCN the outer contour of the wall is without indentations [79].

### 5.3. Pyogenic Liver Abscess

**Figure 25:**
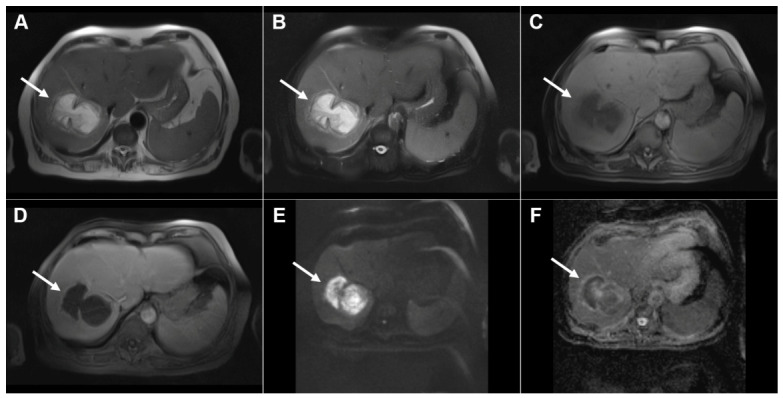
Images of a 79-year-old man with hepatic abscess. Axial T2-weighted (**A**) and T2-weighted fat-suppressed images (**B**) demonstrate a well-defined, lobulated cystic lesion (*arrow*) in liver segment VII with a uniformly thickened wall. Arterial (**C**) and portovenous phase (**D**) images show progressive rim enhancement. Marked diffusion restriction (**E**) is present within the lesion (*arrow*), with a corresponding low signal intensity on the ADC map (**F**), consistent with a hepatic abscess.

Pyogenic hepatic abscess represents localized collections of suppurative material within the liver parenchyma, most commonly caused by Gram-negative bacteria [80]. The characteristic imaging appearance of pyogenic abscess includes central fluid-like pus collection surrounded by a multilayered wall described as the “double target sign” [81]. Contrast-enhanced MRI demonstrates early peripheral enhancement in the arterial phase (ring sign) with persistent enhancement of the wall in delayed phases, while the necrotic central part remains avascular, allowing differentiation from malignant lesions which have progressive stromal enhancement [81]. Diffusion restriction is also a pathognomonic finding in pyogenic liver abscess reflecting the high viscosity and cellularity of purulent contents (Figure 25) [82].

Another typical appearance is the “cluster sign” representing the confluence of multiple small microabscesses into a larger collection [81]. The perilesional liver parenchyma may demonstrate transient segmental enhancement in the arterial phase due to edema and inflammation [83]. The presence of known intra-abdominal biliary infections or previous hepatobiliary procedures, in combination with characteristic imaging features, favors the diagnosis of pyogenic hepatic abscess.

**Figure 26:**
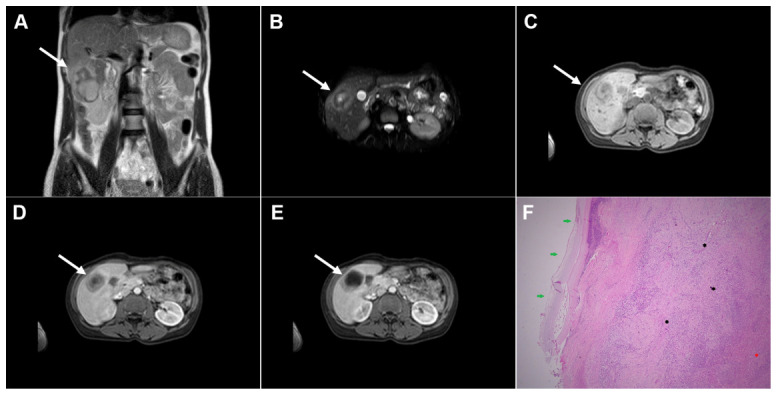
Infected hepatic echinococcal cyst in a 38-year-old woman. Coronal T2-weighted (**A**) and axial T2-weighted fat-suppressed images (**B**) demonstrate a cystic lesion (*arrow*) with a thickened wall and heterogeneous internal content in liver segment V. A focal mural protrusion at the cranial aspect of the cyst wall extends into the adjacent liver parenchyma, corresponding to the presumed site of initial rupture. The layered appearance of the cyst wall (*arrow*) can be noted in the native T1-weighted fat-suppressed image (**C**). In portovenous phase images at two consecutive sections, a targetoid appearance of the wall (*arrows*) is depicted (**D**,**E**). Hematoxylin and eosin (H&E) staining (**F**) shows the inner surface of the pseudocyst lined by acellular, lamellar, eosinophilic, hyalinized material consistent with a laminated (cuticular) membrane, a feature compatible with a hydatid cyst (*green arrows*); fibro-inflammatory cyst wall (*black asterisks*); and adjacent liver parenchyma (*red asterisk*); original magnification ×400.

With regard to differential diagnosis with cystic echinococcosis presenting as a unilocular cyst, CE1 is particularly relevant in endemic regions. Findings of a thick, enhancing wall with a non-enhancing necrotic center suggest the diagnosis of hepatic abscess. In contrast to liver abscess, echinococcal cysts typically do not show an enhancing rim and surrounding hyperemia unless secondarily infected. Furthermore, restricted diffusion is a hallmark of abscesses, whereas uncomplicated echinococcal cysts rarely demonstrate diffusion restriction [82]. The clinical context provides additional clues for differential diagnosis. Acute presentation with fever, leukocytosis, or right upper quadrant pain favors hepatic abscess, whereas CE typically has an indolent course. Nevertheless, in cases of infected echinococcal cysts, the precise differentiation from liver abscess might be difficult, especially in ultrasound or CT examination [84]. In such cases, the visualization of daughter cysts or a detached membrane, if present within a thick-walled cystic lesion in MRI, indicates an infected echinococcal cyst (Figure 26) [85]. In addition, serological tests may support the diagnosis of echinococcosis when imaging findings are equivocal. Given that management strategies differ substantially, accurate differentiation between infected CE and pyogenic liver abscess is very important. While hepatic abscesses usually require systemic antimicrobial therapy, often combined with percutaneous drainage, echinococcal cysts may be managed with antihelminthic therapy or surgical intervention [84].

### 5.4. Cystic Metastases

**Figure 27:**
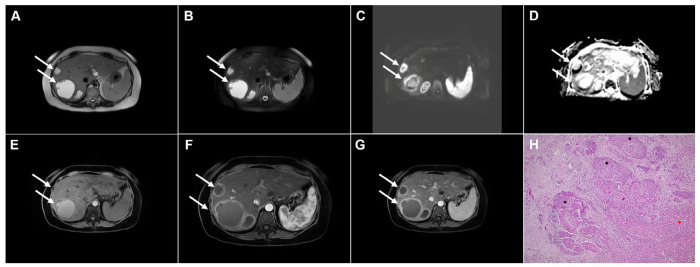
Cystic squamous cell hepatic metastases from cervical carcinoma in a 59-year-old woman. Axial T2-weighted (**A**) and T2-weighted fat-suppressed (**B**) images demonstrate multiple cystic liver lesions (*arrows*) with irregular walls, which show marked diffusion restriction on DWI sequences (**C**) and hypointensity on the ADC map (**D**). The lesion content (*arrows*) is hyperintense in the T1-weighted fat-suppressed image (**E**), consistent with internal hemorrhage. Arterial (**F**) and portovenous phase (**G**) images show intense peripheral (ring-like) enhancement of the lesions, consistent with cystic metastases (*arrows*). Hematoxylin and eosin (H&E) staining (**H**) shows metastasis of a well to moderately differentiated squamous cell carcinoma (*black asterisks*) and the surrounding liver parenchyma (*red asterisks*); original magnification ×400.

**Figure 28:**
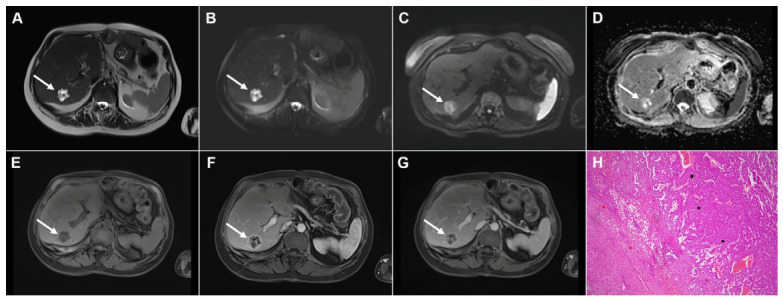
Cystic neuroendocrine hepatic metastases from pancreatic neuroendocrine tumor in a 75-year-old woman. Axial T2-weighted (**A**) and T2-weighted fat-suppressed (**B**) images demonstrate a multilocular cystic liver lesion (*arrow*) with irregular internal septations located in liver segment VI. The lesion (*arrow*) shows mild restricted diffusion on DWI (**C**) and on the corresponding ADC map (**D**). The lesion (*arrow*) is hypointense in the T1-weighted fat-suppressed image (**E**). Rim enhancement can be observed in arterial (**F**) and portovenous (**G**) phase images, with opacification of internal septations (*arrow*). Hematoxylin and eosin (H&E) staining (**H**) shows a well-differentiated neuroendocrine tumor (*black asterisks*), and the surrounding liver parenchyma with features of focal macrovesicular change in hepatocytes (*red asterisks*); original magnification ×400.

Cystic liver metastases are relatively uncommon and are most often seen in neuroendocrine tumors, malignant melanoma, sarcomas, gastrointestinal tumors, and squamous cell carcinomas [85,86]. The cystic appearance of metastases results from central ischemic necrosis or intratumoral hemorrhage due to rapid tumor growth outpacing its vascular supply [85,86]. In patients with a known primary malignancy, the diagnosis is usually straightforward based on clinical context. However, cystic metastases may occasionally be the initial presentation of an occult primary tumor. Cystic metastases typically present as multiple, well-circumscribed cystic lesions with variable wall thickness and internal septations (Figure 27 and Figure 28) [87].

The differential diagnosis of cystic metastases and cystic echinococcosis can be challenging, particularly in endemic regions. Key imaging features favoring cystic metastases include the presence of multiple lesions with irregular or thickened walls, heterogeneous internal architecture, and absence of the characteristic laminated membrane or daughter cysts seen in CE [88]. After contrast administration, cystic metastases may show irregular peripheral or septal enhancement, occasionally with nodular areas reflecting residual viable tumor tissue, whereas echinococcal cysts lack enhancing mural nodules [86]. Additionally, in diffusion-weighted imaging, cystic metastases can show restricted diffusion along the enhancing rim due to high cellularity, in contrast to CE cysts (Figure 28) [86]. CE4 and CE5 lesions may appear as heterogeneous lesions, resembling necrotic or degenerated cystic metastases in T2-weighted images, but their avascularity is usually sufficient for correct diagnosis [32]. Calcifications, seen in CT, can aid in differentiation, as they typically involve the cyst wall in CE. In contrast, calcifications in metastases are often irregular or scattered within the lesion and are most commonly seen in treated metastases [89]. A history of primary malignancy, rapid lesion growth, and the presence of additional metastatic lesions elsewhere support the diagnosis of cystic metastases. Conversely, epidemiological risk factors for echinococcosis, the presence of daughter cysts, and characteristic peripheral wall calcifications favor the diagnosis of CE.

### 5.5. Solitary Necrotic Nodule

**Figure 29:**
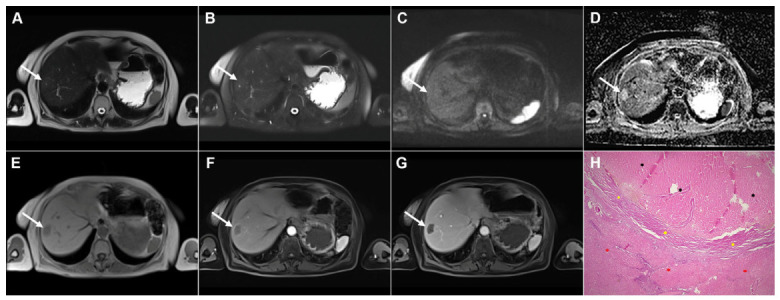
Solitary necrotic nodule in a 77-year-old woman. Axial T2-weighted (**A**) and T2-weighted fat-suppressed (**B**) images demonstrate a mildly hyperintense lesion (*arrow*) in liver segment VIII, with a small intralesional focus of high signal intensity corresponding to central necrosis. Mild hyperintensity (*arrow*) can be observed in the diffusion-weighted image (**C**), with corresponding high signal in the ADC map (**D**), indicating the absence of diffusion restriction. The lesion (*arrow*) appears hypointense in the T1-weighted fat-suppressed image (**E**), with subtle peripheral ring enhancement in arterial (**F**) and portovenous phase (**G**) images. Hematoxylin and eosin (H&E) staining (**H**) shows a solitary necrotic nodule (*black asterisks*), a thin fibrous wall (*yellow asterisks*) that clearly demarcates the described nodule from the surrounding liver parenchyma (*red asterisks*); original magnification ×400.

A solitary necrotic nodule (SNN) of the liver is an uncommon benign lesion, histologically characterized by a necrotic core surrounded by a dense hyalinized fibrous capsule [90,91]. It is generally considered to represent an organized form or “burnt-out phase” of different benign lesions, which may include parasitic infection, abscess, or a thrombosed hemangioma [92]. Typically, SNNs show a low signal intensity in T1-weighted images and, in most cases, hypointensity in T2-weighted images. As the evolution of degenerative changes impacts appearance in T2-weighted images, areas of T2-weighted hyperintensity might also be observed [92]. In post-contrast images, a solitary necrotic nodule is avascular, although the fibrous capsule may demonstrate mild peripheral enhancement in delayed-phase imaging (Figure 29) [92,93]. Due to its solid appearance in T2-weighted images and peripheral enhancement, SNNs usually mimic malignant hepatic lesions, such as metastases or intrahepatic cholangiocarcinoma [94,95]. Moreover, given their heterogeneous appearance in T2-weighted images with predominant hypointensity and avascularity, they might masquerade as the CE4 or CE5 stage of hydatid cysts [95]. Additionally, both lesions display no diffusion restriction, in contrast to malignant mimickers. Ultrasound or CT can further assist in the differential diagnosis by revealing calcifications in the cyst wall in cases of inactive CE, whereas calcifications in SNNs, if present, are typically located within the necrotic core and may reflect prior infection or hemorrhage [92]. Taking into account that both entities are benign lesions, accurate differentiation between SNNs and inactive stages of CE is not clinically important. Recognizing the benign nature of both entities is essential to avoid unnecessary surgical intervention, as conservative management with radiological follow-up is usually sufficient once malignancy has been excluded.

## 6. Differential Diagnosis of Alveolar Echinococcosis

### 6.1. Cholangiocarcinoma

**Figure 30:**
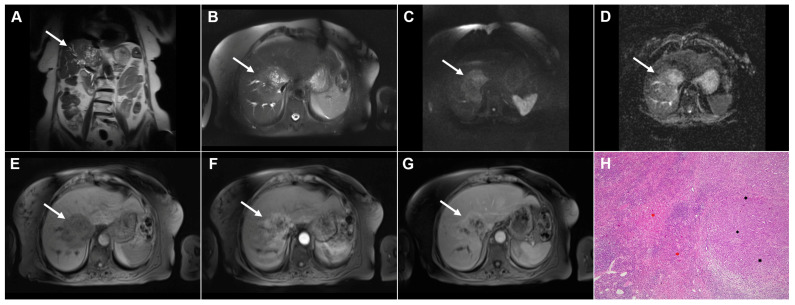
Intrahepatic mass-forming cholangiocarcinoma in a 78-year-old man. Coronal T2-weighted (**A**) and axial T2-weighted fat-suppressed (**B**) images show a lobulated, heterogeneously hyperintense tumor (*arrow*) centrally located in liver segments VII and VIII. Note an irregular eccentric hyperintense component consistent with a mucin-rich area, as well as small hyperintense foci surrounding the lesion, consistent with ectasia of small bile ducts. In the diffusion-weighted image (**C**) and the corresponding ADC map (**D**), the lesion (*arrow*) demonstrates restricted diffusion. The lesion (*arrow*) appears hypointense in the T1-weighted image (**E**), with irregular peripheral and central enhancement in the arterial (**F**) and progressive enhancement in the portovenous phase (**G**). Note the presence of perilesional biliary dilatation. Hematoxylin and eosin (H&E) staining (**H**) shows moderately to poorly differentiated cholangiocellular carcinoma (*black asterisks*) and the surrounding liver parenchyma (*red asterisks*); original magnification ×400.

Intrahepatic cholangiocarcinoma (ICC) is the second most common primary liver tumor, presenting as a mass-forming lesion in 80% of cases [96]. The typical imaging findings favoring the diagnosis of ICC are the presence of a lobular heterogeneous lesion associated with capsular retraction, proximal biliary dilatation, and progressive delayed enhancement [97]. Since AE presents similarly as an irregular infiltrative lesion composed of dense fibrotic tissue, misdiagnosis with ICC might occur []. Furthermore, AE might also show capsular retraction and proximal biliary dilatation. Stojkovic et al. [98] demonstrated in their study, which included 80 patients with AE, that the most common malignant misdiagnosis was intrahepatic cholangiocarcinoma. The presence of multiple small cystic vesicles in T2-weighted images in conjunction with a solid component is a pathognomonic finding for AE and should raise suspicion of this rare entity [52]. However, multiple small cysts might be seen in ICC due to cystic dilatation of small bile ducts at the tumor periphery (Figure 30).

**Figure 31:**
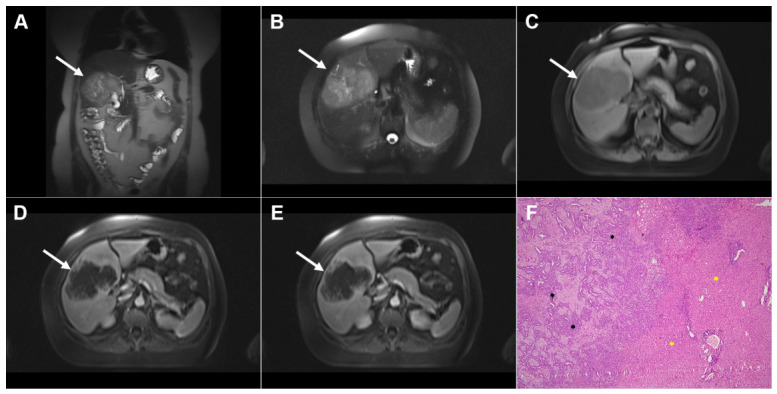
Mass-forming intrahepatic cholangiocarcinoma in a 79-year-old man. An irregular, heterogeneously hyperintense lesion (*arrow*) can be seen in the coronal T2-weighted image (**A**) and axial T2-weighted fat-suppressed image (**B**) in liver segments IVb and V, associated with perilesional biliary dilatation. In the native T1-weighted fat-suppressed image (**C**), the lesion (*arrow*) appears hypointense, with subtle peripheral ring enhancement in the arterial phase (**D**) and no appreciable enhancement in the portovenous phase (**E**). Hematoxylin and eosin (H&E) staining (**F**) shows well- to moderately differentiated cholangiocellular carcinoma (*black asterisks*), as well as normal liver parenchyma (*yellow asterisks*); original magnification ×400.

The central portions of the AE may show necrotic or liquefied areas, while necrosis is rarely seen in ICC. Unlike ICC, in AE, contrast enhancement is minimal, heterogeneous, or peripheral, and the absence of the typical progressive delayed enhancement strongly favors the diagnosis of AE [52]. Nevertheless, the central parts of ICC may remain avascular in dynamic contrast-enhanced MRI due to dense fibrosis or a rich mucin content imitating AE (Figure 31). The use of hepatobiliary contrast agents may aid in distinguishing between AE and ICC, since ICC may present a cloud-like appearance in the hepatobiliary phase with central hyperintensity and peripheral hypointensity, while AE is uniformly hypointense [99]. Furthermore, the detection of calcifications as markedly hypointense foci in MRI is an additional feature indicating AE [53]. Another imaging feature useful for differentiation between these two entities is the morphology of hepatic involvement, which in AE tends to be more geographic and infiltrative, sometimes crossing anatomical boundaries, in contrast to the more regular and concentric mass-forming pattern of cholangiocarcinoma [53]. Regarding differential diagnosis of AE and ICC, DWI does not provide additional information, since both lesions display diffusion restriction at the periphery with a variable signal intensity in the central parts depending on the presence of necrosis in cases of AE or mucin in ICC [54]. Although distinguishing between ICC and AE might be hardly possible in certain cases, since major hepatic resection is the gold-standard treatment for both entities, preoperative differentiation is not mandatory in operable patients. On the other hand, in inoperable cases, percutaneous biopsy is advised.

### 6.2. Liver Metastases

**Figure 32:**
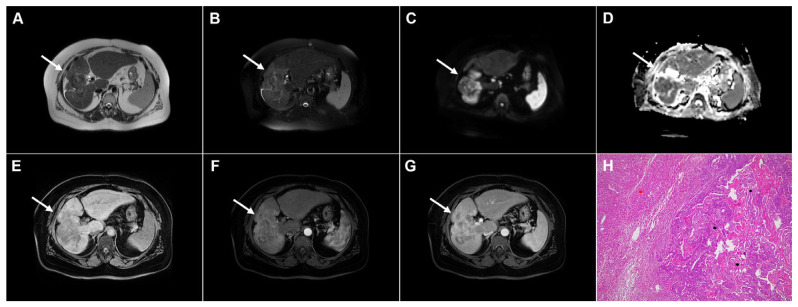
Solitary hypovascular colorectal liver metastasis in a 54-year-old woman. A heterogeneous, slightly hyperintense lobulated lesion (*arrow*) with capsular retraction can be seen in liver segments V and VI in axial T2-weighted (**A**) and T2-weighted fat-suppressed images (**B**). In the diffusion-weighted image (**C**) and the corresponding ADC map (**D**), the lesion (*arrow*) demonstrates restricted diffusion. The tumor (*arrow*) appears hypointense in the T1-weighted fat-suppressed image (**E**), with heterogeneous peripheral and central opacification in the arterial phase image (**F**) and progressive enhancement in the portovenous phase image (**G**). Hematoxylin and eosin (H&E) staining shows metastasis of colorectal carcinoma accompanied by foci of tumor necrosis (*black asterisks*) and the surrounding liver parenchyma (*red asterisks*); original magnification ×400 (**H**).

Hepatic metastases constitute one of the most common liver lesions, and due to their heterogeneity in imaging presentation, these lesions might mimic many other benign and malignant focal liver lesions [100]. Although the diagnosis is not difficult in cases when the primary tumor is already known, in patients without a known primary malignancy, differential diagnosis from various infectious and granulomatous conditions may be challenging [101,102]. Large lesions, particularly colorectal metastases, may exhibit a pronounced internal desmoplastic reaction and a dense fibrous stroma, leading to capsular retraction and a low signal intensity in T2-weighted imaging (Figure 32) [100].

**Figure 33:**
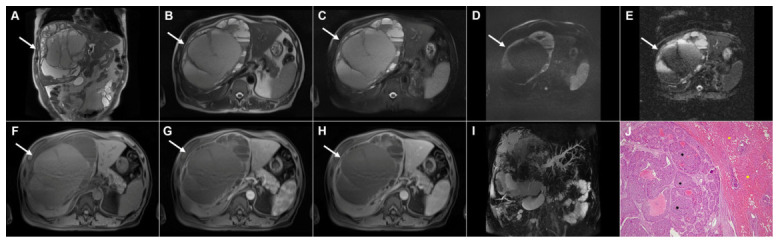
Neuroendocrine tumor metastasis in a 53-year-old man from an unknown primary site. Coronal (**A**), axial T2-weighted (**B**), and axial T2-weighted fat-suppressed images (**C**) demonstrate a large multilocular cystic lesion (*arrows*) in the right hepatic lobe, with multiple internal septations and heterogeneous internal content. Note the presence of fluid–fluid levels, indicating intralesional hemorrhage. In the diffusion-weighted image (**D**) and the corresponding ADC map (**E**), marked diffusion restriction is confined to the hemorrhagic portion of the lesion (*arrow*). The hemorrhagic component appears hyperintense in the T1-weighted fat-suppressed image (*arrow*) (**F**). Mild enhancement of the cyst wall and internal septations can be observed in the arterial phase image (**G**), becoming more pronounced in the portovenous phase image (*arrow*) (**H**). 3D MRCP (**I**) shows a large cystic lesion occupying the central part of the liver, with a mass effect on the biliary confluence and subsequent biliary dilatation in the left lobe. Hematoxylin and eosin (H&E) staining (**J**) reveals areas of neuroendocrine tumor with partially nested, pseudoglandular, and pseudocystic architectural patterns (*black asterisks*), as well as surrounding liver parenchyma (*yellow asterisks*); original magnification ×400.

Additionally, biliary dilatation proximal to the lesion may be observed. Similar imaging characteristics can also be seen in alveolar echinococcosis, which, like metastases, often presents as multiple hepatic lesions. In both lesions, zones of central necrosis are often seen (Figure 33). Given the relative rarity of AE, it may frequently be misdiagnosed as metastatic disease [103].

The presence of microcystic internal structure with numerous tiny T2-hyperintense vesicles embedded in T2-weighted hypointense fibrotic tissue, along with markedly reduced or absent enhancement, should indicate another diagnosis, rather than metastases [53]. If CT is performed, the pattern of calcifications might facilitate establishing the correct diagnosis [8]. Calcifications may occur in metastases, particularly mucinous adenocarcinoma, but are usually less extensive and less characteristic than the coarse, punctate, or confluent calcifications that commonly appear in AE [104]. Moreover, metastases typically displace rather than infiltrate surrounding liver tissue, while AE demonstrates infiltrative growth with potential involvement of bile ducts or vasculature. The slow progression, minimal enhancement, and distinctive vesicular architecture of AE in MRI, combined with appropriate epidemiologic and serologic context, aid in reliably distinguishing it from metastatic disease.

### 6.3. Hepatic Epithelioid Hemangioendothelioma

**Figure 34:**
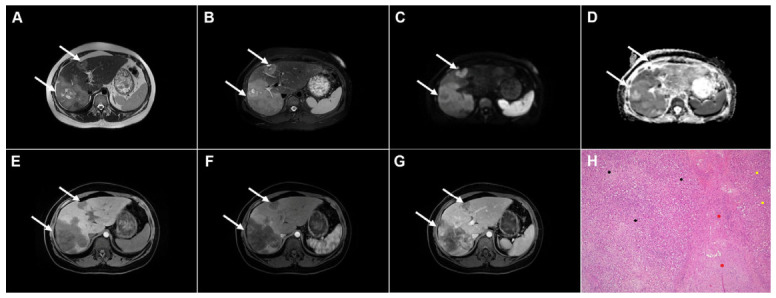
Hepatic epithelioid hemangioendothelioma in a 43-year-old women. Axial T2-weighted (**A**) and T2-weighted FS images (**B**) show two heterogeneously hyperintense lesions (*arrows*) in liver segments VII and IVB. Note also the slight capsular retraction associated with the lesion in liver segment IV. The tumor (*arrows*) shows a high signal intensity in the diffusion-weighted image (**C**), with low ADC values in the corresponding ADC map (**D**). The axial T1-weighted FS image (**E**) shows that the lesions are hypointense (*arrows*). Only subtle perilesional enhancement can be noted in the arterial phase (**F**), with gradual centripetal enhancement in the portovenous phase image (*arrows*) (**G**). Hematoxylin and eosin (H&E) staining (**H**) showed areas of hemangioendothelioma corresponding to a vascular neoplasm composed of anastomosing vascular channels lined by atypical endothelial cells with an epithelioid morphology, embedded in a fibrous stroma (*black asterisks*), a fibrous wall (*red asterisks*) demarcating the tumor from the surrounding liver parenchyma (*yellow asterisks*); original magnification ×400.

Hepatic epithelioid hemangioendothelioma (HEH) is a rare malignant liver tumor which typically presents as multiple, subcapsular lesions associated with capsular retraction and a tendency to coalesce over time [105,106]. With regard to tumor vascularity, Kim et al. [107] have reported that HEH shows a targetoid appearance in a postcontrast study, reflecting its vascular composition and fibrous stroma (Figure 34). The “lollipop” sign is also described as a characteristic finding for HEHE, presenting as a peripheral hepatic lesion with a central necrotic or hypointense “head” and a hepatic vein entering and abruptly terminating at the lesion’s edge, forming the “stick” of the lollipop in contrast-enhanced CT or MRI—a feature typically absent in the geographic infiltrative pattern of AE [108,109]. When compared with alveolar echinococcosis, both lesions exhibit slow growth and demonstrate capsular retraction. Nevertheless, certain imaging findings can facilitate differentiation. AE lesions usually appear as irregular, infiltrative masses with multiple small cystic components and areas of central necrosis often without significant enhancement [53]. Moreover, AE may involve the biliary tree or hilar structures, sometimes leading to biliary obstruction [52]. In contrast, HEH lesions, although they are also peripheral and capable of causing capsular retraction, have more defined margins, a characteristic enhancement pattern, and lack the calcifications and multiloculated cystic structure typical of AE [110,111]. From a therapeutic perspective, the standard of care for both AE and solitary HEH is complete surgical resection with curative intent, rendering preoperative differential diagnosis less critical unless radical surgical resection is not possible when percutaneous biopsy is recommended.

### 6.4. Liver Granulomatosis

**Figure 35:**
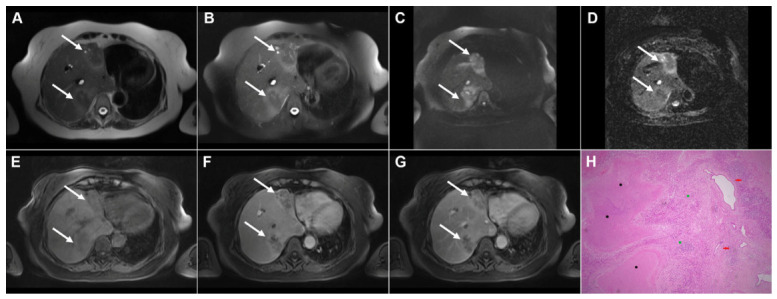
Confluent liver granulomas in a 72-year-old woman. Axial T2-weighted (**A**) and T2-weighted fat-suppressed (**B**) images show two ill-defined heterogeneous lesions (*arrows*) located in liver segments II and VII, with a central hypointense component and peripheral hyperintensity. The lesions (*arrows*) demonstrate restricted diffusion on DWI (**C**), with a low signal intensity in the corresponding ADC map (**D**). In axial T1-weighted fat-suppressed images (**E**), the lesions (*arrows*) appear hypointense, with slight enhancement in the arterial phase (**F**) and progressive central enhancement in the portovenous phase (**G**). Hematoxylin and eosin (H&E) staining (**H**) reveals large, partially confluent necrotizing granulomas (*green asterisks*), fibrous inflammation surrounding the granulomas (*black asterisks*), and intrahepatic bile ducts (*red arrows*); original magnification ×400.

Liver granulomas are defined as collections of epithelioid histiocytes, lymphocytes, and occasionally multinucleated giant cells and may be the result of a variety of conditions, usually infectious or non-infectious conditions, including tuberculosis, sarcoidosis, schistosomiasis, brucellosis, fungal infections, primary biliary cholangitis, drug-induced reactions, and certain autoimmune or idiopathic diseases [112,113]. Granulomas arise through persistent inflammation, typically when the immune system is unable to eliminate a foreign material, microbial pathogen or ongoing antigenic stimulus [114]. When lesions are multiple and there is a relevant clinical history of sarcoidosis, mycobacterial, or fungal infection, the diagnosis is usually unequivocal based on imaging findings. However, in cases where clinical data are scarce, the differentiation from other hepatic lesions can be challenging as there are no specific imaging findings which could suggest a diagnosis of liver granulomatosis [115]. The presence of calcifications and absent or slight post-contrast enhancement may occasionally lead to misdiagnosis with alveolar echinococcosis (Figure 35). Furthermore, both entities show an indolent clinical course with slow growth and non-specific clinical symptoms. However, liver granulomas do not exhibit the infiltrative behavior seen in AE, and in T2-weighted images, these lesions usually have a uniformly or heterogeneously low signal intensity, whereas multiple T2-weighted hyperintense vesicles are characteristic of AE. Additionally, if areas of liquefactive necrosis are present, a diagnosis of AE should be favored. Histopathological examination remains the gold standard for definitive diagnosis, as radiological findings alone are often non-specific [112,115].

### 6.5. Sclerosing Hemangioma

**Figure 36:**
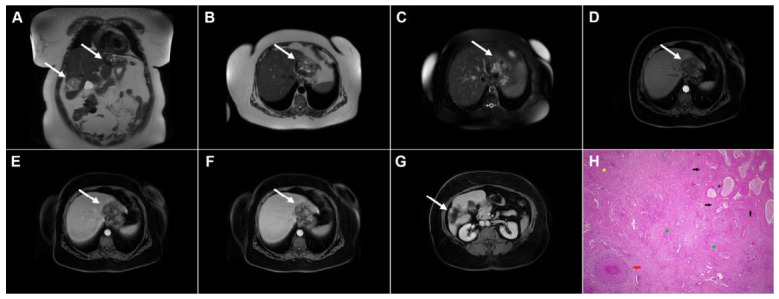
Sclerosing hemangioma in a 33-year-old woman. In the coronal T2-weighted image (**A**), two heterogeneous lesions (*arrows*) can be seen in liver segments II and VI. Axial T2-weighted (**B**) and T2-weighted fat-suppressed (**C**) images demonstrate intermixed hyperintense and hypointense areas within the lesion (*arrow*) in liver segment II. In the T1-weighted fat-suppressed image (**D**), the lesion appears hypointense (*arrow*), with small patchy areas of intense enhancement in the arterial phase (**E**), which become more pronounced in the portovenous phase (**F**). A similar enhancement pattern can be observed in the lesion (*arrow*) located in liver segment VI (**G**). Hematoxylin and eosin (H&E) staining (**H**) reveals a sclerosing hemangioma with components of hemangioma (*black arrows*), a necrotizing granuloma (*red arrow*), chronic fibrous inflammation (*green asterisks*), and liver parenchyma (*yellow asterisk*); original magnification ×400.

Sclerosing hemangioma of the liver develops as a consequence of degenerative changes in liver hemangioma [116]. Minor hemorrhage or thrombosis of vascular channels and subsequent fibrosis and hyalinization within a hemangioma may initiate a fibrotic process leading to the development of sclerosing hemangioma [116]. If vascular stroma is completely replaced by fibrosis, these hemangiomas are called sclerosed hemangiomas [117]. As a consequence, the typical T2-weighted hyperintensity seen in hemangiomas, known as a “light bulb” sign, is absent. Instead, sclerosing hemangiomas display a heterogeneous, mildly to moderately hyperintense T2-weighted signal intensity, interspersed with hypointense fibrotic bands (Figure 36) [116]. Dynamic contrast-enhanced MRI displays different types of enhancement depending on the amount of fibrosis, ranging from a thick rim of arterial hyperenhancement with progressive non-nodular incomplete enhancement to centripetal patchy enhancement with partial unenhanced areas [117,118]. Diffusion-weighted imaging may show a mixed pattern, where sclerosed regions demonstrate relatively restricted diffusion, whereas residual vascular elements show higher ADC values [119]. Due to its heterogeneous appearance in T2-weighted images, AE might mimic sclerosing hemangioma. However, the multivesicular architecture of AE is absent in sclerosing hemangioma, which contains no true cystic vesicles and maintains a more organized internal structure. AE often demonstrates minimal, irregular, or purely peripheral enhancement, while in most cases of sclerosing hemangioma, a typical post-contrast enhancement pattern of a hemangioma in the form of centripetal nodular progressive enhancement is preserved at least in part of the lesion and should be carefully sought in imaging [120,121]. In addition, these parts of the lesion correspond to areas of preserved T2-weighted hyperintensity. While AE shows geographic and infiltrative growth patterns with encasement of vascular and biliary structures, sclerosing hemangioma remains non-infiltrative and well circumscribed [119]. The aforementioned differences in imaging presentation are usually sufficient for an accurate differential diagnosis.

## 7. Conclusions

Due to increasing global travel and population movements, liver echinococcosis has spread from endemic areas to previously non-endemic regions and now represents a significant diagnostic and clinical challenge in everyday clinical practice. Characteristic imaging features, such as the “cyst-in-cyst” appearance in cystic echinococcosis and the infiltrative, multivesicular pattern in alveolar echinococcosis, usually allow accurate differentiation from other cystic and neoplastic liver lesions. However, in addition to these typical presentations, numerous atypical forms may mimic other cystic and solid hepatic lesions, particularly when echinococcosis is not initially considered in the differential diagnosis. In clinical practice, a stepwise approach should be applied in the evaluation of suspected hepatic echinococcosis. In patients with a relevant epidemiological history and ultrasound-detected suspicious hepatic lesions, hepatic echinococcosis should be considered. While typical ultrasound findings may be sufficient for diagnosis in typical cases, MRI is particularly useful in atypical or inconclusive cases for further lesion characterization. Serological tests can support the diagnosis when positive, although negative results do not reliably exclude the disease. Thus, MRI findings should be interpreted in conjunction with epidemiological, clinical, and serological data to guide final diagnostic decision-making. Awareness of atypical presentations and potential complications, including biliary obstruction, rupture, and vascular involvement, is crucial for accurate diagnosis and appropriate management. In such cases, MRI provides valuable complementary information for diagnosis, staging, and treatment planning for both CE and AE. Careful assessment of lesion morphology, signal characteristics, and enhancement patterns offers diagnostic clues that complement clinical and serological findings, thereby supporting timely and effective therapeutic decisions.

## Data Availability

The original contributions presented in this study are included in the article. Further inquiries can be directed to the corresponding author..

## Abbreviations

The following abbreviations are used in this manuscript:

CE	Cystic Echinococcosis
AE	Alveolar Echinococcosis
MRI	Magnetic Resonance Imaging
WHO-IWGE	World Health Organization—Informal Working Group on Echinococcosis
CT	Computed Tomography
CL	Cystic Lesion
ADC	Apparent Diffusion Coefficient
MCN	Mucinous Cystic Neoplasm
SNN	Solitary Necrotic Nodule
ICC	Intrahepatic Cholangiocarcinoma
MRCP	Magnetic Resonance Cholangiopancreatography
HEH	Hepatic Epithelioid Hemangioendothelioma
